# In temperate Europe, fire is already here: The case of The Netherlands

**DOI:** 10.1007/s13280-023-01960-y

**Published:** 2024-02-05

**Authors:** Cathelijne R. Stoof, Edwin Kok, Adrián Cardil Forradellas, Margreet J. E. van Marle

**Affiliations:** 1 Department of Environmental Sciences, Wageningen University, PO Box 47, 6700 AA Wageningen, The Netherlands; 2 Nederlands Instituut Publieke Veiligheid, PO Box 7010, 6801 HA Arnhem, The Netherlands; 3 Technosylva Inc, La Jolla, CA USA; 4 Joint Research Unit CTFC - AGROTECNIO - CERCA, Solsona, Spain; 5 Department of Crop and Forest Sciences, University of Lleida, Lleida, Spain; 6 Deltares, PO Box 177, 2600 MH Delft, The Netherlands

**Keywords:** Area burned, Emerging fire regions, The Netherlands, Wildfire causes, Wildfire occurrence, Wildfire statistics

## Abstract

Landscape fires are usually not associated with temperate Europe, yet not all temperate countries record statistics indicating that actual risks remain unknown. Here we introduce new wildfire statistics for The Netherlands, and summarize significant events and fatalities. The period 2017–2022 saw 611 wildfires and 405 ha burned per year, which Copernicus’ European Forest Fire Information System satellite data vastly underestimate. Fires burned more heathland than forest, were small (mean fire size 1.5 ha), were caused by people, and often burned simultaneously, in Spring and in Summer drought. Suppression, restoration and traffic delays cost 3 M€ year^−1^. Dozens of significant events illustrate fire has never been away and has major societal impact amidst grave concerns for firefighter safety. Since 1833, 31 fatalities were reported. A legal framework is needed to ensure continuity of recordkeeping, as the core foundation of integrated fire management, to create a baseline for climate change, and to fulfill international reporting requirements.

**Supplementary Information:**

The online version contains supplementary material available at 10.1007/s13280-023-01960-y.

## Introduction

Landscape fires are most often associated with Mediterranean areas and less so with temperate regions. While it is commonly accepted that climate change is expected to increase wildfire risk in temperate regions (Veira et al. 2016; Arnell et al. 2021), few people realize that fire is already there. Fires in temperate climates are often much smaller than in Mediterranean areas and because of this remain undetected by satellites (San-Miguel-Ayanz et al. 2019), while ground-based records of fire statistics are not systematically collected. An example is The Netherlands, a country famous for its flood risk and water management, which significant fire history is unknown to many, even inside the country itself. Despite high-quality data collection up until 1994 (Boosten et al. 2009), The Netherlands does not officially keep statistics of landscape fires, and it is not the only country that lacks solid data on their fire management challenge (San-Miguel-Ayanz et al. 2022). Yet accurate information about when, where and how landscape fires occur is essential to ensure adequate awareness and preparedness for these fires, both now and in the future.

**Fig. 1:**
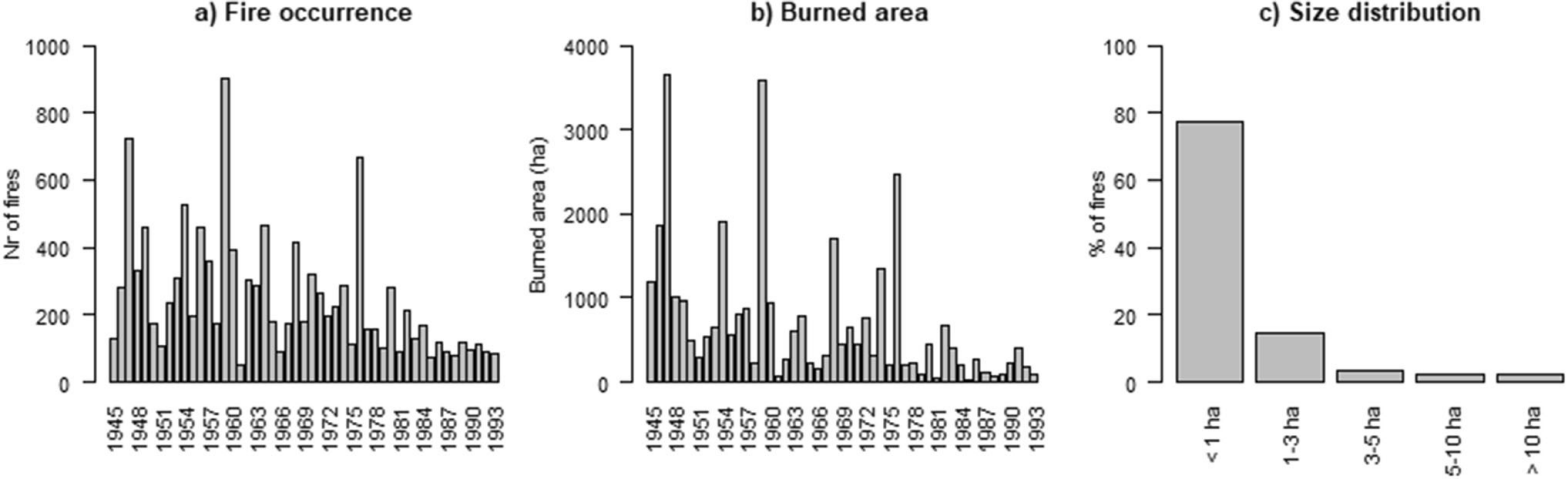
Historic patterns of **a** annual fire occurrence and **b** annual area burned (1945–1993) and **c** distribution of fire size (1978–1993) in The Netherlands. *Source* IKC (1995)

Landscape fires are abundant across Northwest Europe (e.g. Fig. 1a, b), and in normal years these fires are concentrated in Spring (April–June; de Jong et al. 2016; Tapia et al. 2022) when vegetation is dry as the sap flow has not recommenced after winter. In dry windy weather, these Spring fires can spread fast, particularly when flammable grasses such as Molinia spp. are present. Because green leaves contain a lot of moisture, summer fires after the green-up in Spring are an exception in temperate Europe but do occur after severe drought causing wilting and die-off of vegetation and intense drying of organic layers, such as in 2018 (San-Miguel-Ayanz et al. 2019; Tapia et al. 2022). Compared to Mediterranean regions, temperate Northwest Europe not only experiences the timing of the peak fire season earlier in the year, but the region also differs in terms of the vegetation type most affected by fire. Fires do not predominantly occur in forests but rather in heathlands and other open habitat (IKC 1995; Gazzard et al. 2016). Another major difference is fire size: because of considerable landscape fragmentation but also likely because of short travel times for fire services, fires tend to be much smaller than in typical fire countries. In The Netherlands for instance, 97% of all landscape fires between 1978 and 1993 was smaller than 10 ha (Fig. 1c). While these small fires may seem irrelevant to those from traditionally fire prone countries, they form a considerable challenge and it is important to acknowledge that landscape fires are a relatively new risk in temperate Europe, particularly when compared to other threats like floods and windstorms. Mitigation of wildfire risk is often still absent in planning and design, due to low awareness and preparedness amongst major stakeholders and the general public. Moreover, fire services in this region tend to be specialized in urban fires rather than landscape fires, such as is the case in the UK, Belgium and The Netherlands (Depicker et al. 2020; Stoof et al. 2020; Belcher et al. 2021). Within this context, accurate information on where, when, how and why fires burn is essential for baseline information to assess effects of climate change, and to increase awareness of fire as an emerging risk in these often densely populated regions. Wildfire statistics are also essential for cost–benefit analysis of fire risk reduction strategies, which de Hoop et al. (2022) highlighted as a tool to create (political) will to take such measures. And finally, while it is known that people cause the vast majority of fires (FAO 2007; Keeley & Syphard 2018; Belcher et al. 2021; San-Miguel-Ayanz et al. 2022) and good methods exist for assessing fire origin and cause (NWCG 2016), fire cause remains largely understudied and unknown (San-Miguel-Ayanz et al. 2022). As such, important information is missing to design effective prevention and awareness campaigns.

In Europe, landscape fire statistics including fire occurrence, size and cause are collected by individual countries, collated in the Copernicus European Forest Fire Information System (EFFIS), and discussed by the EU Expert Group of Forest Fires (EGFF). Both EFFIS and EGFF were launched in 1998 and originally focused on Southern Europe, and now represent 43 countries across Europe, the Middle East and North Africa (San-Miguel-Ayanz et al. 2022). While EFFIS and EGFF both refer to forest fires in their name, many contributing countries also report fires in non-forest areas, such as in shrubland and agricultural land. While participation in EGFF and data contribution from European Member States is voluntary, data on area burned is essential for the accurate reporting of greenhouse gas emissions. Since The Netherlands stopped its ‘forest and nature fire’ statistics collection in 1994 (Box 1), the country’s fire emission reporting (Arets et al. 2021) has been based on historical data from a period (1980–1992) that is characterized by very mild fire years (IKC 1995). Accurate reporting of fire occurrence and fire size is therefore not only crucial from a public safety perspective but also from the perspective of emissions reporting. It should therefore be highlighted that The Netherlands is not the only country in Europe without official landscape fire statistics, six other European countries (Denmark, Luxembourg, Moldova, Belarus, Andorra, and Liechtenstein) are not currently part of the EFFIS network. Germany is part of EFFIS but limits its statistics to fires in forests and does not consider fires in open habitat such as heathlands or agricultural land (San-Miguel-Ayanz et al. 2022), and while the UK does have extensive landscape fire statistics there are issues with underreporting of fires managed by land managers, counting of wildfires that also impact structures, and UK-wide analysis and publication (personal communication Gareth Clay, Rob Gazzard). As such, attention to and improvements of landscape fire statistics reporting is a broader issue than just in The Netherlands.

Our goal was to create (1) a database of current landscape fires in The Netherlands regardless of vegetation type and size, (2) an overview of significant historical fires in The Netherlands, and (3) an overview of landscape fire related fatalities in The Netherlands, that are not centrally registered. We describe the development of a new wildfire statistics database as well as the 6 years of data now collected (2017–2022). We then use these data to characterize the recent Dutch fire regime and place this in context a 52-year long time series of significant fires in The Netherlands (1970–2022) and a 189-year long timeseries (1833–2022) of landscape fire fatalities.

**Table:** Box 1 History of ‘forest and nature fire’ statistics collection in The Netherlands

The Netherlands had a strong record of well-kept fire statistics from 1922 organized by various land management agencies that systematically recorded wildfire occurrence, area burned, vegetation type, presumed cause, location and size. These dedicated records were halted in 1994 because of decreasing fire numbers (Fig. 1a, b), along with ongoing conversion of coniferous forest to less fire-prone deciduous forest (Boosten et al. 2009)—despite most fires occurring in open habitat like heathland, not in forest (IKC 1995). Stopping the dedicated recordkeeping suggests that wildfire was not considered an issue anymore. European legislation at the time did require countries to have forest fire prevention plans aimed at reducing the number of forest fires (and thus requiring statistics to demonstrate the achievements), but these plans were only mandatory for regions with medium–high fire risk, which countries themselves could classify (European Commission 1992). As such, this European law did not hinder The Netherlands to stop collecting dedicated landscape fire statistics. In 1994, the Dutch registration of wildfires was transferred from the forest service to the Central Bureau of Statistics (CBS) that is in charge of the indoor and outdoor fire statistics. CBS did not count wildfires as a separate category, but instead as part of all outdoor fires: ‘fires in the open air that do generally not involve buildings’, a category that includes fires in trashcans and vehicles, in addition to fires in vegetation. This strongly inflated the number of fires recorded, rising from 50 to 900 *wild*fires (Fig. 1; IKC 1995) to ≈28,000 *outdoor* fires per year (CBS 2015; period 1985–2013), two orders of magnitude higher. This major difference can be explained by the fact that (1) wildfires only form a small part of all outdoor fires recorded by CBS; (2) CBS data is not validated, but also includes false alarms, training exercises, and multiple counts of the same incident; and (3) CBS does not consider fires originally not labeled as outdoor fires but that required deployment of additional fire engines and post-fire assessment indicating that it concerned a wildfire. As such, the inflation of fire numbers past 1994 left The Netherlands without dedicated and reliable wildfire statistics. In 2002, 8 years after the collection of dedicated ‘forest and nature fire statistics’ was halted, the only forest fire policy in the country was abolished (SER 2002), completing the fire prevention paradox. This policy required land owners to maintain fire breaks and a specified road density to ensure forest accessibility to the fire service, while also prescribing a maximum height of forest slash piles and windrows. While this policy did not prescribe management regarding vertical connectivity of fuels, it did have requirements to avoid the use of ‘*Pinus* and other flammable species’ in forests adjoining heathland, natural grassland or other flammable vegetation (Bosschap 1962). As outlined in the retraction announcement (SER 2002), the policy was abolished because (1) it only considered forest, not other vegetation types; (2) forest fire was increasingly considered less of a problem, with ‘fire suppression being so effective that larger fires practically do not occur anymore’, and (3) the Dutch forest was considered increasingly less flammable because ‘flammable forest stands are not planted anymore, small-scale forest management, an increasing proportion of deciduous trees in the crown layer, and the development of a vital shrub layer’ (SER 2002). Despite a few local initiatives, there is currently no national, regional or local policy instrument regarding wildfire risk or impact prevention, reduction or mitigation. In 2017, The Netherlands joined EFFIS and EGFF after the first and second author of this present study started an informal transdisciplinary collaboration to collect wildfire statistics, the methods and analyses of which are presented here.	

## Materials and methods

### Study area

**Fig. 2:**
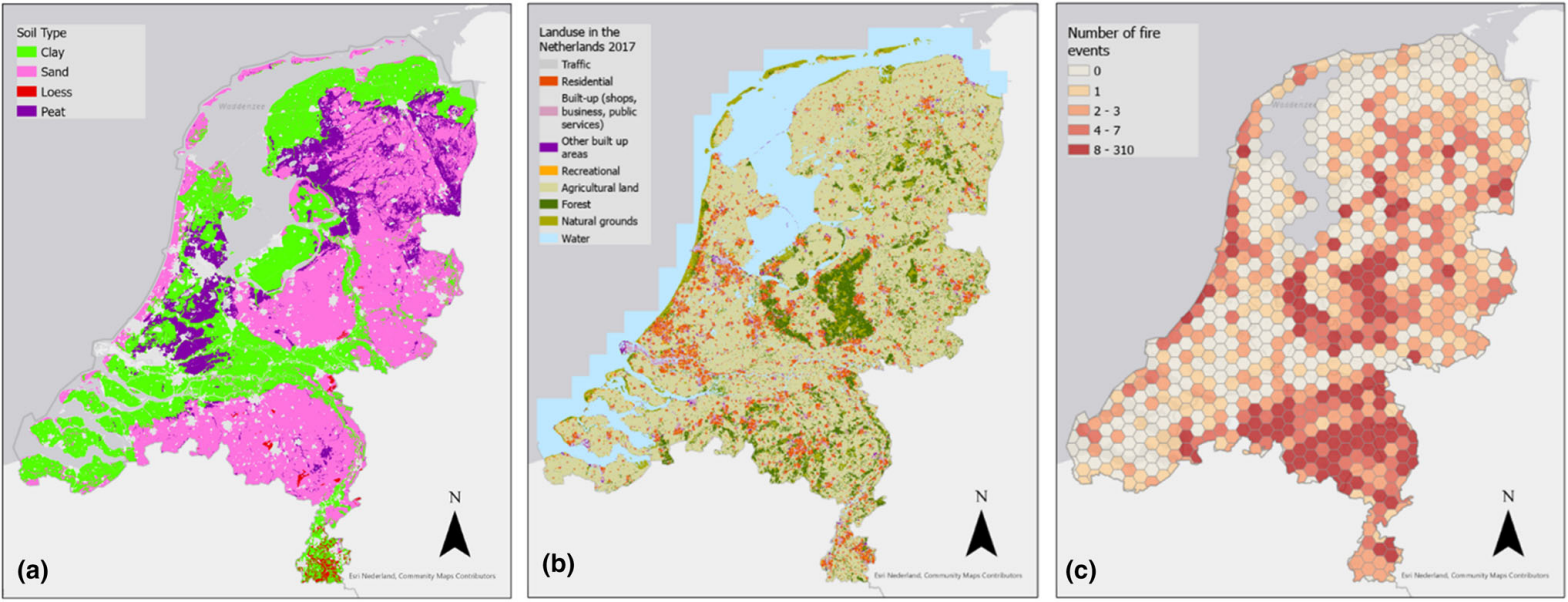
Soil map (**a**, source: Basisregistratie Ondergrond) and land use map in 2017 (**b**, source: Central Bureau of Statistics), and **c**) spatial distribution of landscape fires from 2017 to 2022 (number of fires) aggregated per 50 km^2^ (hexagon colors)

The Netherlands is located in Northwest Europe, with elevation ranging from 6.8 m below to 322 m above mean sea level (Rijkswaterstaat 2022). Climate is temperate with an annual average temperature of 10.5 °C (0.7 °C in February to 23.1 °C in July); mean annual rainfall is 855 mm (KNMI 2022). While Dutch water management traditionally focused on draining water from the landscape as quickly as possible, recent drought years have increased attention for the importance of retaining water in the sandy upland landscape to mitigate effects of drought. The Netherlands has ≈18 million inhabitants (522 people km^−2^; CBS 2022). Soils in the Dutch delta are sandy, clayey and peat soils (Fig. 2a), and is a major control of land cover (Fig. 2b). The total natural land cover is 586 kha (363 kha forest, 54 kha heathland, 101 kha semi-natural grassland and 35 kha marshland; CLO 2017). The Dutch landscape is highly fragmented with small patch sizes, interspersed by a dense network of roads, railways, bicycle paths and walking tracks.

### Current landscape fire statistics

We counted all vegetation fires regardless of their size or the vegetation type they burn in. Data collection commenced in 2017 as an informal collaboration between the first and second author. We here report the first 6 years of data (2017–2022).

Fires are counted as wildfires when they meet either of the following two criteria:

1. The incident classification is “wildfire” (*natuurbrand*) or a related term (e.g. *heidebrand,* “heathland fire”*; veenbrand,* “peatland fire”*, bosbrand,* “forest fire”; *duinbrand,* “dune fire”, or *rietbrand,* “reed fire”); or
2. The incident is not classified as such but additional resources were sent to the fire and afterwards information (from safety regions or news agencies) indicated the fire occurred in vegetation.

Roadside fires (*bermbrand*) are counted when they meet the second criteria (see Appendix S1).

Because all fires that meet these criteria above are counted regardless of their size, fires that burn only 10 or 100 m^2^ are also included in our database, to allow users to use their own size cut-off during any post-processing of data. Stored data includes the approximate location, dates and times the incident was reported, fire size, cause and confidence level of the cause, any media source consulted (website URL), resources requested (fire engines, water trucks, aerial means, drone assessment, handcrew, wildfire advisor), and any comments. Note that the information on resources requested considers the resources that were *called* to provide support—whether or not they were actually *used* during the fire is currently not recorded. Fire size and vegetation type are estimated from photo and video material or in the field, and fire cause classified following Camia et al. (2013). For details and quality assurance, see Appendix S1; data are provided by Stoof et al. (2023).

### Costs of recent Dutch landscape fires

With the information currently in the database, an estimate of direct suppression and restoration costs was made. Fire suppression costs were based on Kok et al. (2023), who use standard values for fires < 1 ha (base scenario, 2 k€), 1–100 ha (regional scenario, 52 k€) and > 100 ha (supra-regional scenario 1 M€). We adjusted for the typically lower costs of 1–10 hectare fires, assuming 3 h of fire suppression at 7 k€ hour^−1^ (personal communication Klaas Noorland), leading to an estimate of 21 k€ per fire. Considering post-fire restoration costs (incl. fencing, signposts, trails, replanting), Kok et al. (2023) reported values of 900–1200 € ha^−1^ for the Meinweg and Deurnese Peel fires, and 6000 € ha^−1^ for the 2011 Kalmthoutse Heide fire. Here we assume the lowest value of 900 € ha^−1^ as a conservative estimate for all fires regardless of their size class.

Indirect costs due to the loss of functioning of highway infrastructure were estimated for fires that occurred along highways between 2017 and 2022 (n = 74), focusing on events that required scaling up of fire service resources. This is an underestimation of all next-to-road fires, as highway authorities for instance counted 228 vegetation fires in 2018, with only 22 requiring scaling up of fire service resources and thereby appearing in our dataset. The cost calculation is therefore again conservative. Following Bles et al. (2020), we assume half of the road’s capacity to remain available during a fire, resulting in 50% of users to experience delays due to traffic jams and 50% delays due to detours, and that only one direction (the nearest) is affected. Mean duration of verge fires reported by highway authorities is 40 min (Bles et al. 2020); we calculated losses for 1-h closures per fire, likely still an underestimation given that this calculation considered the larger next-to-road fires only.

### Historical record of significant fires

To create an overview of significant fires between 1970 and 2022 we complemented information from internal fire service archives (available from 2009) with a search of historical media reports sourced from Digibron^1^ and NOS^2^. This search was targeted at larger fires (> 50 ha) as well as fires of any size that had significant social, economic or environmental impact, such as accidents, victims, damaged buildings, evacuations, and/or a large number of fire service resources deployed. Using fire size information provided in the media reports as well as in formal reports (IKC 1970, 1976; Roerink & Arets 2016; Stoof et al. 2020) and satellite analysis (Appendix S1), the top 5 largest wildfires since 1970 was determined.

### Fatalities

A list of landscape fire fatalities was created from the Dutch firefighter monument (*Brandweermonument*), national fire service documentation center (*Nationaal Brandweerdocumentatiecentrum*, NBDC), internet and colleagues. The national firefighter monument (3) records firefighter deaths in line of duty since World War II (5 May 1945). Three NBDC lists were screened: fire service fatalities from 1635 to 2022, fatal fires from 1807 to 2011, and a detailed list from 2001 to 2014. Fires were considered as landscape fires if they met the criteria listed in “Study area” section. Two cases where fire burned in a forest but the fatalities were the result of an external cause (a plane crash not related to the forest fire, and a murder case involving a burning object in the forest) were excluded from analysis.

## Results

### The recent Dutch fire regime

**Fig. 3:**
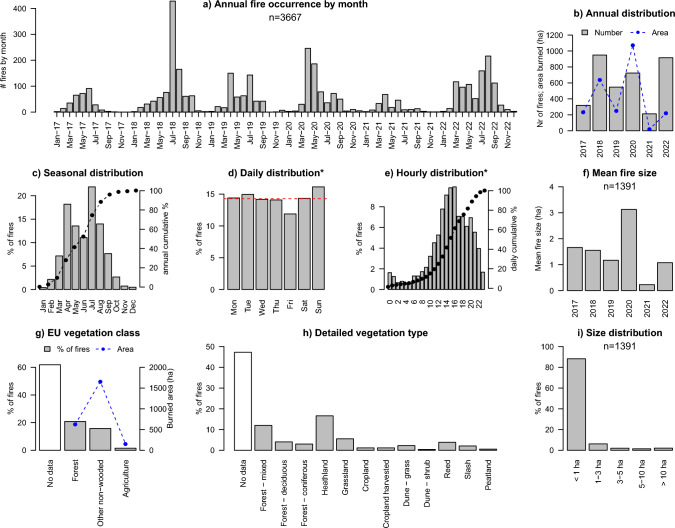
Temporal characteristics and other metrics of recent landscape fires in The Netherlands (2017–2022): number of fires by month and year (**a**), year (**b**), month (**c**), weekday (**d**), and time of day (**e**, time that fire was reported), mean fire size (**f**), vegetation type affected following EU classification (**g**), detailed vegetation type (**h**), and fire size distribution (**i**). Blue lines in (**b**) and (**g**) indicate a conservative estimate of burned area. Data represented include all fires (n = 3667, incl. on military terrain) in all plots except for subplot d and e that consider non-military fires only (n = 3096). Burned area data considers fires for which these data were available (n = 1390; plot **b**, **f**, **g**, **i**). The fires on the two military shooting ranges are the direct result of shooting practice which is conducted year round. These fires are therefore included in the annual and seasonal overviews, but because the shooting practice is done on set moments during the week and day these fires are excluded from weekday and hourly overviews so as to avoid bias. Because of the considerable gaps in burned area reporting we do not include here the seasonal, weekly or hourly burned area data

Between 2017 and 2022, The Netherlands saw 3667 landscape fires in total, particularly during the drought years of 2018 and 2022 (Fig. 3a, b) and on sandy soils (east, south and along the coast, Fig. 2a, c). All fires except two were contained on the same day, only the 2020 fires in the Deurnese Peel (4 days) and in de Meinweg (2 days) burned overnight and into the next days. The annual number of fires ranged from 212 in 2021 to 949 in 2018 (Fig. 3b), with an average of 611 ± 306 fires per year. 15.6% of these fires occurred on military terrain. The seasonal distribution shows that fires predominantly occurred not only in Spring (April-June) but also in Summer (July, August; Fig. 3c). Zooming in on fires not related to military shooting practice, 16.1% of fires occurred on Sundays (Fig. 3d), significantly more than could be expected if fires were evenly distributed across the week (p = 0.002, $\chi$^2^ goodness-of-fit test). 69% of all non-military fires started during daytime (7:00–19:00 h), and 51% started in the afternoons (13:00–19:00 h; Fig. 3e). Fires often happened concurrently: there were 40 days that saw 15 fires or more, with 31 fires the maximum recorded in a given day. Such multi-occurrence days were often consecutive, like in Summer 2018, Spring 2019, and Summer 2022. In 2020, there were five consecutive days in April 2020 that saw 94 fires total (Table S1), including two multi-day transboundary fires (Deurnese Peel, Meinweg).

Fire size information was available for 38% of fires (n = 1390), with a total of 2432 hectares reportedly burned between 2017 and 2022. Burned area follows a slightly different trend to fire occurrence. While 2021 saw the smallest area burned (18 ha), 2020 rather than 2018 or 2022 was the year with the largest reported burned area (1073 ha; Fig. 3b), due to the contribution of the fires in the Deurnese Peel (710 ha) and de Meinweg (≈200 ha) in 2020. The Deurnese Peel fire was also the largest fire that occurred in the study period. Figure 3i shows that the distribution of fire size is highly skewed, with 88% of fires < 1 ha, and only 2% > 10 ha. Considering only the fires for which size information was included (n = 1391), mean fire size was 1.5 ± 1.0 hectares (Fig. 3f).

Information regarding fire size as well as the vegetation type burned was available for 62% of fires (n = 2269). Of all these fires, there were fewer fires in forest/woodland (21%) than in non-wooded land (16%) and only 1.5% occurred in agricultural land (Fig. 3g). Zooming in on the more detailed vegetation classes identified relevant to the Dutch landscape (available for n = 1934 fires, 53% of total), 17% occurred in heathland and 6% occurred in grassland (Fig. 3h). Interestingly, out of the 86 agricultural fires that occurred, 43 fires occurred in *harvested* cropland. The area reportedly burned was highest for heathland (866 ha) and peatland (716 ha).

**Fig. 4:**
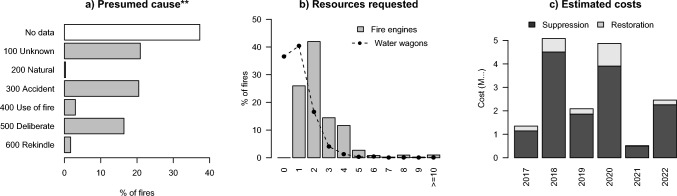
Presumed fire cause (**a**, n = 3667), number of fire engines and water tenders requested (**b**, for n = 3583 and n = 2270 fires, respectively), and estimate of suppression and restoration costs (**c**). **Note that while the fire cause classification in (**a**) follows the EU classification system, fire cause was only informally assessed, hence these data consider presumed cause only

While fire cause was informally assessed in 2300 fires, official fire cause investigations were very rare: only 16 out of the total of 3667 fires were officially investigated for fire origin and cause, a mere 0.4%. Because of insufficient evidence, cause was determined for none of these few officially investigated fires. Information on presumed fire cause of the majority of fires can therefore only be derived from informal assessment (Fig. 4a), suggesting humans are the predominant cause of fire in The Netherlands, with only 10 fires (0.3%) being caused by lightning. Informal assessments furthermore suggest that 601 fires (16%) were deliberately caused, and 753 fires (21%) were accidental. The 669 fires that occurred due to shooting practice on military terrain are classified as part of this latter category. Three percent of fires were a result of fire use (e.g. negligence, vegetation burning, recreation, fireworks) and 2% was due to a rekindle (Fig. 4a). Only one fire in the database is assumed to be caused by cigarettes. While this is very valuable information, it is important to note that these were all informal assessments that have not been investigated following official procedures (i.e. by trained wildfire cause investigators).

### Costs and resources requested

Moving from fire occurrence and burned area to ancillary information of these fires, Fig. 4b and c show an overview of the resources requested, a conservative estimate of suppression and restoration costs. The majority of fires saw 1–2 fire engines and no water tenders requested (Fig. 4b); most resources were requested for the Deurnese Peel and Meinweg fires (100 fire engines each, 7 and 4 water tenders, respectively). In total, 8953 fire engines and 3522 water tenders were requested over the 6-year study period. Furthermore, the following additional resources were used: drone team (53 times), handcrew (18 times), specialist wildfire advisors (17 times), wildfire spread model and aerial suppression (both 9 times).

The conservative estimate of suppression and restoration costs (Fig. 4c) and amounts to a 6-year total of 16.4 M€, of which 87% went to suppression. This amounted to 2.7 ± 1.9 M€ year^−1^ average, with mean annual suppression and restoration costs being 2.4 ± 1.6 and 0.4 ± 0.4 M€ year^−1^, respectively. The costs of delays due to highway disruptions was 70 to 540 k€ year^−1^, with an average of 297 ± 185 k€ year^−1^ and a 6-year total of 1.8 M€.

### Historical record of significant fires and their impacts

**Fig. 5:**
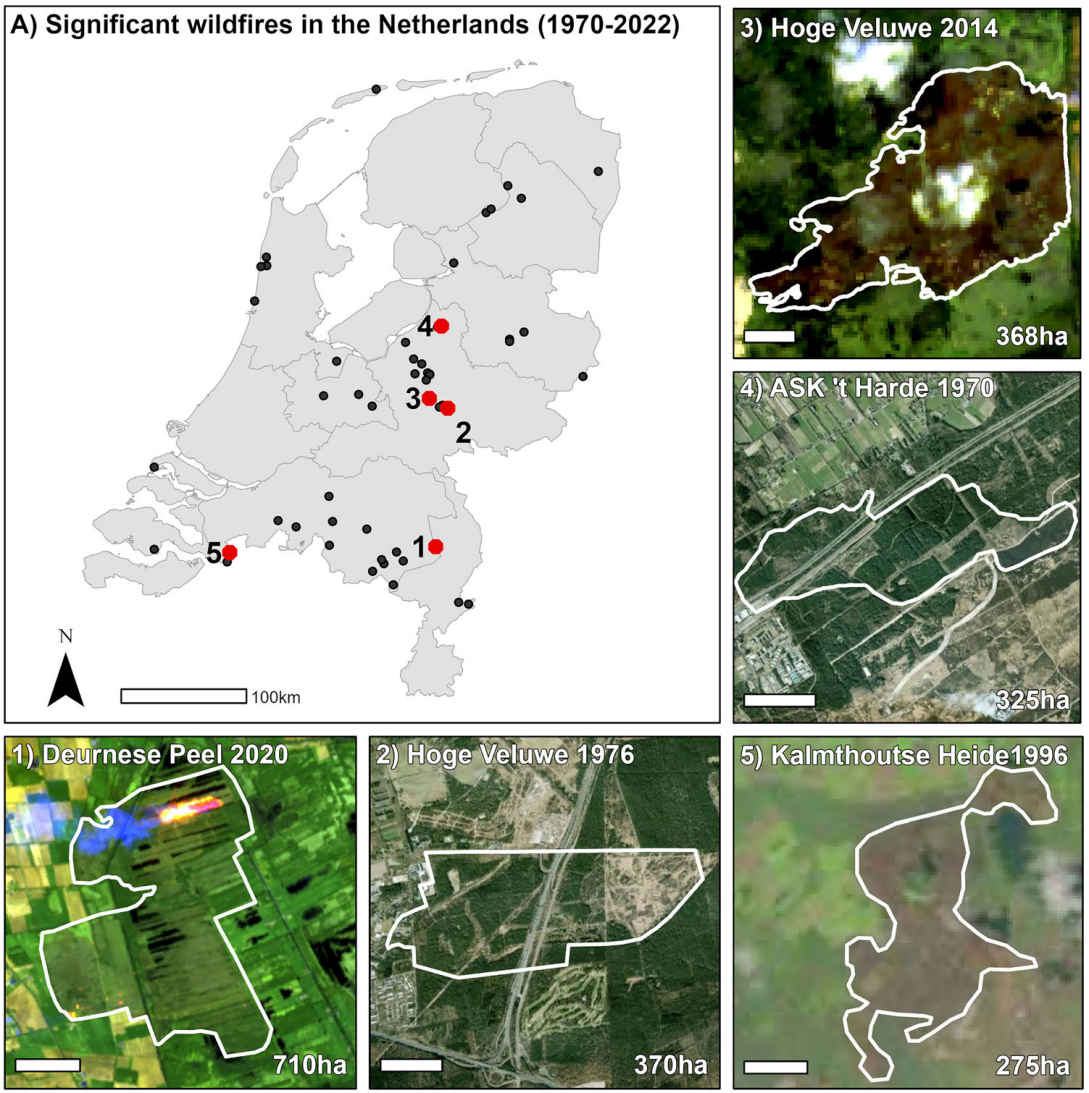
Spatial distribution of significant wildfires between 1970 and 2022 (**a**), with red points representing the location of the largest fires shown in detail: (1) Deurnese Peel, 20 April 2020 (Stoof et al. 2020); (2) Hoge Veluwe, 7 July 1976 (IKC 1976; Lavèn, 1976); (3) Hoge Veluwe, 20 April 2014 (Roerink and Arets 2016); (4) ASK ‘t Harde, 18 June 1970 (De Stentor 2010; IKC 1970); (5) Kalmthoutse Heide, 21 April 1996 (Landsat analysis). See Appendix S1 for methods and background image sources. The white bar in plots 1–5 represents 1 km

The search of historical fires in The Netherlands created from internal archives and news media resulted in a list of 65 significant fires that occurred between 1970 and 2022. The earliest in this record was the 1970 fire near’t Harde that burned 325 hectares and destroyed six farms and two villas, with people fleeing in chaos and panic (Broekman 1985; IKC 1970). The 1.4 M€ 1976 fire near Arnhem, which burned 370 ha (IKC 1976), is perhaps the best known wildfire in Dutch history. This fire jumped roads and threatened homes and people, as well as Burger’s Zoo where staff were preparing guns to shoot the zoo’s predators should the fire come closer (Table 1). A range of these significant fires occurred in a transboundary context, on provincial and national borders (Fig. 5a). Despite typically lasting only a few hours and burning at most a few hundred hectares (Fig. 5), the reported impact of the events was considerable and included traffic disruptions (blockages of highways, trainlines, ships), evacuations of thousands of people, smoke impacts, road accidents and homes burned. The fires have resulted in evacuation of a range of campsites [Loonse and Drunense Duinen 1996; Aamsveen 2011; Wedde 2018; Wateren 2018], homes [Veenhuizen 2015; Deurnese Peel 2020], villages and towns [Schoorl 2009; Bergen 2009; Bergen aan Zee 2010; Herkenbosch 2020], elderly homes [Schoorl 2009; Deurne 2020], a National Park with its world famous art museum and Van Gogh paintings [Hoge Veluwe 2014], a youth prison and a homeless shelter [Mastbos 2014], residents, holiday makers and animals. These significant fires furthermore threatened additional villages, campsites, a refugee center and prisons. Smoke impacts include those on people close to the fire itself [Meinweg and Deurnese Peel 2020] as well as on towns and cities tens of kilometers away from the fires [Kalmthoutse Heide 1996 causing issues in The Hague, Malpie 2017; Moergestel 2020]. Three fatalities were reported between 1970 and 2022, and only a small number of personal injuries.

**Table 1:** Significant fires since 1970 including their impacts (see Table S2 for sources)

When	Where	What
18 Jun 1970	ASK	Fire caused during shooting practice on military terrain, rapidly spread to 't Harde village, fanned by strong winds. Jumped highway A28, railway and national road. Six farms and two villas burned down, all with thatched roofs. People had to flee in chaos and panic; 1 injured. Reports of fire fighters running for their lives
7 Jul 1976	Hoge Veluwe	Fire started in a forest near Rozendaalse Veld, fanned westwards by strong winds, burning a total of 7 h. It jumped the Apeldoornseweg road and rapidly spread to the village of Schaarsbergen and highway A12, approaching the suburbs of Arnhem where the Burgers Zoo was preparing guns to shoot their predators. 3000 firefighters, including from Germany and large numbers of volunteers helped stop the fire. The army was called in, also to support with mop up that lasted two weeks. Despite the interregional collaboration through the Veluws bosbrandcomité, there were major communication barriers because the various fire services involved used different communication networks, that were also incomplete. No personal injuries were reported, but two fire engines got stuck and were badly burned and two military tanks got stuck and were considerably damaged. Several roads were closed for several days, and the total cost was estimated at 1.4 million guilders (635 k€, not corrected for inflation)
8 Jul 1976	Leenderheide	Forest fire that jumped provincial road, very rapid spread, burned a total of 3 h. Involvement of 17 local fire brigades, two from outside the region, DAF and Philips fire brigades, 200 military personnel. Use of a Leopard tank to remove trees to protect a restaurant. Major communication challenges (shortage of radio's, vehicles without call numbers, different brigades not able to reach eachother)
31 May 1977	Best	Small fire in a forest behind a garbage dump, in which WWII shrapnel explodes that kills a volunteer firefighter
12 May 1980	Doldersum	Doldersummerveld; campsite Sonnekamp in Vledder was only barely saved from the flames. Post-fire management included strategic grazing of cows and pony's, creating the 'one of the most beautiful heathlands of Drenthe'
23 May 1980	Herkenbosch	Fire in pine forest during absence of aerial fire watch (spotter planes) because of financial shortages
15 May 1980	Mariapeel	Fanned by strong winds so rapidly that 'fire fighters could only save themselves by jumping into one of the many fens'. Two firefighters treated with burns in hospital. Fire service difficulties to reach the fire because of inaccessibility of the peat bog terrain and the enormous public interest (onlookers). One house evacuated because of dense smoke; fire duration 5.5 h
15 May 1980	Hoog Soeren	Heathland fire; second largest of 15 fires on this day
24 Apr 1982	Arnhemse Heide	Heathland fire; involvement of 23 fire brigades, fire duration 2.5 h. Provincial road N50 (Arnhem-Apeldoorn) blocked for 2 h. Fire suppression complex because of repeatedly shifting wind direction and significant smoke production
28 Apr 1984	Sprengenberg (Hellendoorn)	4-h long fire in forest and heathland, involvement of several hundred fire fighters, 30 fire engines, tens of farmers with water trucks and as many volunteers with branches and spades. Fire hard to suppress: it was hard to find, inaccessible terrain, strong winds shifting directions
18 Jun 1986	Someren	Heathland fire, involvement of 125 firefighters from 6 local fire brigades and the DAF company fire brigade
10 May 1987	Witterveld (Havelte)	4-h heathland fire in military terrain, involvement of four local fire brigades and the fire brigade of the Veenhuizen Prison. Reports of strong, shifting winds, and numerous spot fires due to embers
27 Jul 1989	Beekhuizerzand	Wildfire during which a Cessna spotter plane crashes, killing two people
23 Apr 1994	Sallandse Heuvelrug	4-h fire, fanned by strong southerly winds; involvement of 22 fire brigades, 36 fire engines and 200 firefighters. Despite the creation of three large water reservoirs after an earlier fire in 1984, fire suppression was challenged by a lack of water. The fire started along a bicycle route and burned the most part of the last breeding ground of the endangered black grouse (*Tetrao tetrix*) on the West European mainland. Two important roads were closed, several accidents occurred on the deviation routes (reports of injuries were not listed)
11 Aug 1995	Kootwijk, A1	4-h heathland and forest fire that started on the shoulder of highway A1, jumps the highway and burns on both sides of it. Due to large smoke production a traffic chaos emerges which challenged the fire brigade to reach the fire; no personal injuries. Drivers abandoned their vehicles, which were later towed way by police and fire service. Highway A1 closed for two days; roadside restaurant evacuated; involvement of 150 fire fighters, 22 fire engines, 8 support vehicles
20 Apr 1996	Hooge en Lage Mierde	Fire in Landgoed de Utrecht
20 Apr 1996	Loonse en Drunense Duinen	Fire service advised evacuation of campsite, that was not at risk
21 Apr 1996	Kalmthoutse Heide	Fire in Belgian-Dutch border area, threatened the cross-border village of Putte, collaboration of Dutch and Belgian emergency services (hundreds of firefighters and tens of fire engines). Several firefighters injured after they were trapped by the fire and had to escape through the flames. Evacuation of campsite prevented after fire was stopped in time. Leopard tanks created a firebreak where the fire was halted. Large smoke production with the smell of smoke reported all the way to The Hague (100 km away) where Dutch parliament is seated
20 Apr 1997	Kalmthoutse Heide	Fire in Belgian-Dutch border heathland
28 Apr 1999	Rozendaal	Fire in a heathland along highway A50, involvement of 20 fire engines, and farmers with water trucks usually used to spread liquid manure
13 May 2001	Loonse en Drunense Duinen	Forest fire
17 Apr 2003	ASK	Heathland fire on military terrain, involvement of 4 fire engines from the department of defense, regional support, and first time deployment of military Chinook helicopter with 10.000 L 'bambi-bucket' water drops
25 Aug 2003	Utrecht	Next-to-train-track fire near railway emplacement, blocked train traffic between Utrecht-Leiden, Utrecht-Den Haag and Utrecht-Rotterdam for 1.5 h and affecting thousands of travellers
15 May 2004	Terschelling	Dune fire (grass, deciduous forest) with three core locations on the Wadden island Terschelling, involvement of island fire brigade, 25 firefighters from the mainland, a Cougar and a Chinook helicopter for aerial support. Smell of smoke noticeable on the mainland
6 May 2006	Hoog Soeren	Forest and heathland fire, involvement of > 100 firefighters, strong winds frequently changing direction
9 Jul 2006	Hoge Veluwe	8-h fire in marshland terrain, involvement of 140 firefighters, 20 fire engines, a Chinook helicopter. Suppression activities challenged by the loose soil; no injuries reported. National Park Hoge Veluwe (Deelense Veld)
29 Apr 2007	Ermelo	1.5-h heathland fire, partial closure of provincial road N302 Ermelo-Apeldoorn. Fire initially burned along the road, then spread fanned by strong winds
28 Aug 2009	Schoorl	25-h dune fire turning into a forest fire that threatened the village of Schoorl, causing evacuation of 550 people including a care home, restaurant, hotel, 200 houses, and holiday homes. This fire started at several locations, around 13.00 h, with strong winds (8 Beaufort, 62–74 km hour^−1^) causing rapid spread and numerous spot fires. Reports of firefighters being nearly trapped, and two fire engines driving off the scene leaving their fire hoses behind. Village of Schoorl covered in smoke, 4-km long traffic jam between Bergen and Schoorl. Fire did not stop at a fire break; rainfall helped. Radio TV channel Noord Holland deployed as a disaster-broadcaster. First of a series of ≈100 fires in 2 years, which stopped after a suspect was caught but not convicted because of a lack of evidence
16 Sep 2009	Bergen	Evacuation of 200 people
24 Apr 2009	Hoog Soeren	Heathland fire near Assel, 30 fire engines and tens of firefighters. Railway Apeldoorn-Amersfoort blocked
27 Apr 2009	Wierdense Veld	Heathland fire, involvement of 80 local and regional firefighters
14 Apr 2010	Bergen	Dune fire causing the evacuation of the entire village Bergen aan Zee, 400 residents as well as holiday makers and an animal zoo. All roads to the village blocked to prevent disaster tourism. Involvement of range of regional fire brigades, Red Cross, ambulance services, riot police, aerial support from the department of defense. Strong winds, 7 Beaufort (50–61 km hour^−1^), 750-m wide fire front
2 Jul 2010	Strabrechtse Heide	Heathland fire involving 1700 fire fighters from eight regional fire brigades, > 700 military personnel, Department of Defense, Rijkswaterstaat, Staatsbosbeheer, along with fire service and police. Closure of highway A67 because of smoke until the next morning and ship traffic on the Zuid Willemsvaart canal, for water intake. An inspection report discusses the hard work under challenging conditions (heat, late supply of food and liquids, long duration of shifts), as well as high appreciation for all the work done. The large number of recommendations given include aspects around education, training, prevention, materials, suppression techniques, working conditions and safety. Four people involved in fire suppression incurred smoke-exposure related injuries
20 Apr 2010	Hoog Soeren	3-h grass and forest fire fanned by strong winds, causing fire 'to turn in a circle', involvement of 200 firefighters, 24 fire engines. Smoke visible on long distance; blockage of trainline Apeldoorn-Amersfoort
1 May 2011	Schoorl	Dune fire burning overnight, requiring the deployment of 500 firefighters from a large number of regions; three fire engines did not reach the fire due to a traffic collision near Alkmaar
24 Apr 2011	Fochteloërveen	Fire in a peat bog on the Dutch-German border, burning overnight; involvement of 150 firefighters. Fire spread in the direction of a prison
3 Jun 2011	Aamsveen	Fire in a peat bog on the Dutch-German border, deployment of 350 German and 75 Dutch firefighters; evacuation of campsite; strong winds, inaccessible terrain; no injuries
25 May 2011	Kalmthoutse Heide	Cross-border heathland fire requiring deployment of 400 firefighters and 95 fire engines from The Netherlands and Belgium. Failure of communication systems caused loss of contact with firefighters during the fire; strong winds repeatedly shifted direction. Fire was stopped after rainfall
1 Apr 2012	Radio Kootwijk	3-h fire; > 5 local fire brigades, no evacuations; part of a series of fires in this area
20 Apr 2014	Hoge Veluwe	Fire in National Park Hoge Veluwe burning on Easter Sunday; requiring evacuation of all park visitors as well as evacuation of valuable pieces of art (incl. Van Gogh, Mondriaan, Picasso and Seurat paintings) to the underground fire shelter
17 Apr 2014	Mastbos	Forest fire requiring evacuation of a youth prison and homeless shelter; 20 fire engines, helicopter
13 Mar 2015	Veenhuizen	Fire in peat bog requiring evacuation of several homes; nearby prison did not require evacuation. Fire started at several locations along a bicycle path
17 May 2015	Chaam	Forest fire; 20 fire engines, helicopter. Nearby refugee center did not require evacuation
18 Jul 2015	Leenderheide	Heathland fire, 150 firefighters, concerns over a high voltage powerline failing and smoke hindering highway A67 but winds shifted just in time
22 Jun 2017	Maria Peel	8-h overnight fire in peatbog
7 Jul 2017	Malpie	Heathland fire; ≈100 firefighters, 8 fire engines, water trucks; smoke impacts on nearby companies, recreation, road traffic, bicycle paths
30 Jun 2018	Wedde	Fire started during harvest in wheatfield, adjacent campsite evacuated because of smoke and embers; ambulance post created on campsite to help any camping guests who may have inhalated smoke
7 Aug 2018	Wateren	6-h heathland, forest and wheatfield fire requiring the evacuation of three campsites and a flock of sheep, involving 150 firefighters from three provinces, 16 fire engines, farmers with water supply, Chinook helicopter. Rapid fire spread with spot fires, flames reached campsites up to a few meters. The first fire engine at the scene needs to flee after trying to attack the fire offensively, losing 20 fire hoses. Trauma helicopter deployed; one person treated for smoke inhalation
16 Jul 2018	Heemskerk	3-h dune fire in inaccessible terrain. Because it is expected the fire will spread rapidly a large number of resources is requested, including support from seven regions, a Chinook helicopter, police helicopter for reconnaisance. An NL-alert (automated text message sent to all nearby cellphones) was sent to evacuate hikers and cyclists from the site. Arson is expected as a bottle of lamp oil was found
18 Jul 2018	Drunen	Forest fire in very inaccessible terrain, reports of at least 1 fire engine stuck in of the loose soil. Much groundfire, as well as crownfire; no wind but falling trees as a result of burning humus layers cause very dangerous conditions for firefighters. Fire is ultimately stopped after handcrew deployment
15 Jul 2018	ASK	On a Sunday (when no shooting practice is done), a 3-h heathland fire burns on military terrain, possibly caused by a phosphorus grenade; involvement of 12 fire engines (4 military, 8 civilian from surrounding villages) support trucks, Chinook helicopter. Possibly caused a pyroCb; smoke visible from far away
1 Jul 2018	Budel	Roadside fire start in grass and heather, moving into a forest under strong winds; jumped a 15-m wide fire break (two roads and a ditch). Involvement of 8 fire engines, multiple water tenders, drone, farmer contractors
27 Feb 2018	Deurnese Peel	Two fires in a peat bog, reportedly caused by human activity. Fire service allows fire to burn the area in a controlled way because of inaccessible terrain; smoke production causes hindrance for several weeks
28 Jul 2018	Woudenberg	2-hour wildfire along highway A12 that started as a roadside fire which moved to the other side of the highway using an ecoduct: a vegetated bridge intended for wildlife crossings. Both the highway and the adjacent railway Utrecht-Arnhem were temporarily blocked, and fire crews from across the region supported fire suppression.
8 Apr 2019	Hilversum	Heathland fire that rapidly develops due to strong wind; assistance from neighboring safety region required
10 Apr 2019	Arnhemse Heide	Sixteen fire engines deployed for a heathland fire that rapidly spread due to strong winds. Fire on military terrain
15 May 2019	Leusderheide	Heathland fire on military terrain, 16 fire engines deployed as well as a water tender and a helicopter for reconnaisance. The fire reportedly started on several locations at the same time, and upon first response had already jumped four dirt roads. The smell of smoke was noticeable in the city of Utrecht, 18 km away. Fire bucket operations for aerial fire suppression were arranged but not needed after the fire was quickly controlled
20 Apr 2020	Deurnese Peel	710-ha fire in a Natura2000 peat bog that burned for four days aboveground and 2 months smoldering with reignitions and major smoke impacts on nearby communities and road traffic, including blockage of highway A67 and a six-vehicle car crash on a regional road. No injuries were reported. Resources deployed include 2000 fire fighters from across the region and the country, 4 Chinook helicopters, as well as intensive work by contractors to extuinguish the smoldering peat
20 Apr 2020	De Meinweg	≈200-ha cross-border fire burning from Germany into The Netherlands, burning at the same time as the Deurnese Peel fire. The fire started along a bicycle path, was first spotted by cyclists passing by and jumped a trail that was designed as a 'compartment boundary' in the fire mangement plan. The fire spread erratically and rapidly, at some point against the wind, surprising and nearly trapping German firefighters that had to flee the scene leaving their fire hoses behind. 4200 people in the town of Herkenbosch (incl. residents, a holiday park and a horse riding school) were evacuated because of carbon monoxide concerns at the height of the first corona-pandemic wave, an evacuation that was later assessed as unnecessary. Resources deployed include helicopters, a Leopard tank for the military to create a 40-m wide fire break, as well as several hundred Dutch firefighters and 1600 German firefighters. The fire created significant smoke production, causing reports of smoke and ash coming down in the city of Hasselt (Belgium), 65 km away. Large numbers of onlookers were sent away due to concerns over the spread of COVID
21 Apr 2020	Moergestel	The third major fire burning at the same time in the country, burning for just over 4 h in heathland and forest, rapidly burning 50 ha and causing smoke issues in the city of Tilburg and town of Goirle (10 km away). Belgium fire services provided support. With this third fire, nearly all southern regions have fully deployed all their landscape fire resources
31 Mar 2021	Sint Jansklooster	Controlled reed slash fire in which one person lost their life
14 Apr 2022	Putte	Wildfire in which two soldiers are injured because of smoke intoxication
19 Jul 2022	ASK ’t Harde	60-ha heathland fire on military terrain ASK, caused by shooting practice
9 Aug 2022	Ouddorp	4-ha fire in dune shrubland that threatened a museum, and for which a campsite with 300 guests was proactively evacuated by its owner and national road N57 was closed. A train wagon filled with railway sleepers burned down. Resources deployed include 25 fire trucks, 9 water tenders, a handcrew, and a cougar helicopter for aerial water drops
31 Aug 2022	Mariapeel	41.5-ha wildfire in a peat bog, that was first spotted by birdwatchers at 6.30 am. Resources deployed include a drone team, handcrew, a Chinook and Cougar helicopter. Tactical fire was used to help stop the fire spread. Mop up activities were done by fire service and land managers, lasting until 15 September

Extreme fire behavior in the context of pyrocumulus development was observed in two fires [Strabrecht 2010; ASK 2018]. The list additionally contains frequent reports of the fires spread being fanned by strong winds (often in shifting directions), creating major issues for fire suppression as well as for safety. Many of these fires produced spot fires, with travelling embers causing fires to jump roads, highways railways and canals (Table 1). The data contains a concerning wealth of reports regarding safety threats to fire service personnel. This includes reports of fire fighters running for their lives [‘t Harde 1970; Deurnese Peel 2020], fire spreading so rapidly that *'fire fighters could only save themselves by jumping into one of the many fens*', with two firefighters being treated with burns in hospital [Deurnese Peel 1980, Table 1], several firefighters getting injured after they were trapped by the fire and had to escape through the flames [Kalmthoutse Heide 1996], firefighters being nearly trapped [Schoorl 2009], four people involved in fire suppression incurring smoke-exposure related injuries [Strabrechtse Heide 2010], fleeing fire engines leaving their hoses behind [Schoorl 2009; Meinweg 2020], a fire engine losing 20 fire hoses while needing to flee after attempting direct attack of the fire front [Wateren 2018], falling trees due to burning humus layers [Drunen 2018], and rapid and erratic fire behavior surprising and nearly trapping a crew of German firefighters that had to flee the scene leaving their hoses behind [Meinweg 2020].

### Fatalities

We discovered ten fire events between 1833 and 2022 in which 31 lives were lost (Table 2), including three firefighters in line of duty. Seven fatalities were associated with fire suppression activities, one with fire use. The remaining 23 were civilians that lost their life during large peat fires in Dutch peat mining colonies 1833, 1880 and 1917, with the latter fire causing 18 fatalities. Reports of these peat fires detail people fleeing the flames, having to spend hours in canals to keep themselves safe in the water. They left large numbers of people homeless, and caused major economic impact and loss of employment.

**Table 2:** Fatal landscape fires in The Netherlands

Date	Location	Fatalities	Context	Source
31 Mar 2021	Sint Jansklooster, Overijssel	1	Controlled reed slash fire	https://www.destentor.nl/steenwijkerland/slachtoffer-rietbrand-sint-jansklooster-is-80-jarige-vrouw-uit-steenwijkerland~a26236f5/
27 Jul 1989	Beekhuizerzand, Gelderland	2 (1*)	A Cessna wildfire spotter plane crashed while supporting/accompanying a fire engine on the ground	https://www.digibron.nl/viewer/collectie/Digibron/offset/0/zoekwoord/sportvliegtuigje+verongelukt+brand/id/tag:RD.nl,19890728:newsml_7afee280054ed98be03f42296a812766, https://www.brandweernederland.nl/onderwerpen/brandweermonument/
31 May 1977	Best, Noord-Brabant	1*	A volunteer firefighter died from injuries sustained after being hit by shrapnel from an exploding high-explosive World War II grenade while suppressing a small fire in a forest behind a garbage dump	https://www.brandweernederland.nl/onderwerpen/brandweermonument/
15 Apr 1946	Bergen op Zoom, Noord-Brabant	1*	A volunteer firefighter died from injuries sustained during a vehicle crash while en route to a forest fire in Woensdrecht; three colleagues were injured	https://www.brandweernederland.nl/onderwerpen/brandweermonument/
11 Oct 1939	Hilversum, Noord-Holland	1	The heathland fire engine from fire station Hilversum crashed en route to an exercise; a fire watch (*brandwacht*) died from injuries sustained, nine others were injured	NBDC https://www.korpora.nl/
Summer 1935	Drenthe	1	A person’s clothes caught fire while trying to stop a heathland fire, when fleeing he was caught by the flames when he tripped	Staf and Schenkenberg van Mierop (1936)
21 Apr 1922	Tilburg, Noord-Brabant	1	A farmer collapsed and died while helping suppress a large forest fire	NBDC https://www.korpora.nl/
21 May 1917	Valthermond, Drenthe	18**	Eighteen people lost their life during a large peat fire; other impacts include 120 houses burned, 4 weeks long, large number of hectares turf	Ottens et al. (2017)
1880	Weerdingermond, Drenthe	1	Large peat fire in which a 4-yr old boy was killed, 90 houses burned, hundreds of people were left homeless. Escaped buckwheat burn	https://www.deverhalenvangroningen.nl/alle-verhalen/een-verwoestende-veenbrand-bij-zevenhuizen-1833
11 June 1833	Zevenhuizen, Groningen	4	A large peat fire caused 4 fatalities, 66 houses burned, 5 ships, 1 windmill, hundreds of people homeless, 1.1 million metric tonnes of turf, high unemployment. Reported cause: lightning, ignitions fanned by a southwestern storm	https://www.deverhalenvangroningen.nl/alle-verhalen/een-verwoestende-veenbrand-bij-zevenhuizen-1833

*Fire fighters that lost their life in the line of duty

**Official numbers report 17 fatalities, we here additionally count the baby who was stillborn just hours before her severely injured mother died two days after trying to save her two sons from the fire

## Discussion

### The fire regime of The Netherlands

In project meetings with large teams spanning across Europe, we have regularly heard the comment ‘when fires will happen in the North of Europe with climate change *in the future*’ (our emphasis). The Dutch data shown here show that this future is already here: it already burns. Fire is here (Figs. 2, 3), and the long list of significant fires since 1970 (Table 1) shows it has not really been away. Despite the fact that reporting systems changed between the pre-1994 statistics and the current data collection, the number of fires and area burned are mostly similar (Appendix S2). Estimated area burned averages at 405 ha annually (a conservative estimate given that size is not reported for 62% of fires). Fire numbers are also likely underestimated because small vegetation fires along highways and trainlines are currently not systematically included—this may not affect burned area much, but does affect impact analysis of such fires. Fire analyses is currently complicated because their exact location is unknown: fires are recorded by streetname/municipality, coordinates are not available and fire perimeters are not walked with a GPS or phone app like done elsewhere (personal communication Edgar Nebot), which hinders map-based land cover analyses. For the fires with size information, mean fire size is 1.5 ha (Fig. 3f), in the same order of magnitude as e.g. Austria, Finland, Germany and Sweden and an order of magnitude lower than e.g. France, Greece, Italy, Portugal and Spain (San-Miguel-Ayanz et al. 2022). At 405 ha, the mean annual average burned is 1.6 times higher than the 248 ha Arets et al. (2021) assume for greenhouse gas emissions reporting. Comparing 6-year totals of our ground-based wildfire data with EFFIS (Table 3) shows a stark difference: long term EFFIS records capture 45% of wildfire area burned (1104 out of 2432 ha) and vastly underestimates wildfire occurrence (7 out of 3367 fires), merely 0.2% of all wildfires in The Netherlands. This can be explained by the fact that the EFFIS country totals are a long-term record based on MODIS satellite, that only detects fires above ≈30 ha. At the same time, also the combined MODIS/Sentinel2 product only captures a fraction of Dutch wildfires (6 wildfires and 128 ha in 2022, out of 916 wildfires and 220 ha counted on the ground). It is well known that fire size is highly skewed and that small fires disproportionately contribute to total fire occurrence (de Zea Bermudez et al. 2009). Recent work by Ramo et al. (2021) highlights the impacts of not detecting small fires with coarse-resolution satellite data, leading to sizeable underestimation of area burned and carbon emissions in Africa. In their analysis, fires < 100 hectare accounted for 41% of burned area; for The Netherlands, small wildfires not captured by EFFIS between 2017 and 2022 (99.8% of wildfires) accounted for 55% of wildfire burned area (Table 3).

**Table 3:** Comparison of fire occurrence (number of fires) and area burned between our ground-based data collection (the present paper) and EFFIS estimates obtained from the multi-year country totals for the Netherlands (https://effis.jrc.ec.europa.eu/apps/effis.statistics/estimates/NLD). These EFFIS records are based on fires > 30 ha (personal communication, Pieralberto Maianti)

	Fire occurrence		Area burned	
	Ground-based (this study)	EFFIS	Ground-based (this study)	EFFIS
2008	No data	0	No data	0
2009	No data	0	No data	0
2010	No data	0	No data	0
2011	No data	1	No data	148
2012	No data	0	No data	0
2013	No data	0	No data	0
2014	No data	1	No data	396
2015	No data	0	No data	0
2016	No data	0	No data	0
2017	319	0	232	0
2018	949	3	639	183
2019	547	0	250	0
2020	724	2	1073	822
2021	212	0	18	0
2022	916	2*	220	99*
*2017–2022 total*	*3667*	*7*	*2432*	*1104*
*2017–2022 mean*	*611*	*2*	*405*	*184*

*EFFIS country totals reported 6 fires with a total of 284 ha for 2022. This included four managed burns ≥ 30 ha with a total of 175 ha, and two wildfires with a total of 99 ha. Only the wildfire data is presented in this table

In terms of seasonality, results show indeed a Springtime peak in fire occurrence (Fig. 3c) as expected from historic literature and recent analyses (IKC 1995; de Jong et al. 2016; Cardíl et al. 2023), but also a peak in July that can directly be ascribed to the major 2018 and 2022 Summer droughts (e.g. Philip et al. 2020). The far majority of these fires start during daytime (Fig. 3e) when relative humidity (and thus dead fine fuel moisture) is lower and both wind and human activity are higher, all contributing to a higher probability of fire ignition and spread. The significantly higher fire occurrence on Sundays (Fig. 3d) is not unlike elsewhere in temperate Europe (Albertson et al. 2010) and highlights the human contribution to fire causes, as fires would be spread evenly across the week if causes were only natural. As only 0.4% of all fire causes are officially investigated, there is no hard data on the most prevalent fire causes but informal assessments suggest nearly all fires are anthropogenic, similar to Belgium (Depicker et al. 2020), the UK (Belcher et al. 2021) and elsewhere (FAO 2007). The fact that often these fires happen simultaneously (Table S1) is not surprising given that vegetation senescence, drought and fire danger are processes that are connected. Good international examples of simultaneous fires are for instance the 2017 Chilean fires (Bowman et al. 2019) and Portugal’s October 2017 fires when hundreds of fires occurred during the same day (Ramos et al. 2023). Yet in the Dutch fire service, preparedness for large-scale events is designed for one major incident: ‘multiple large events may occur but it should be accepted that this the exception, and will challenge the fire service’s improvisation skills’ (Brandweer Nederland 2018). Regions are required to provide basic resources to support other regions upon request, but again not based on simultaneous deployment. This lack of preparedness for simultaneous events challenges the capacity to respond to numerous wildfires during periods of high fire danger, making The Netherlands likely to need international support at times that neighbouring countries may also be in need, therefore increasing cross-border vulnerabilities. Regarding the vegetation types affected, most fires and area burned occurred not in forest but in open habitat such as heathland and grassland (Fig. 3g, h), which is similar to e.g. Belgium (Depicker et al. 2020) and Great Britain (Gazzard et al. 2016). Because of this, in our wildfire communication we have shifted our language away from the traditional focus on forest fires to *natuurbranden* (‘nature fires’), a successful shift that has been slowly but steadily taken over by news media as well as stakeholders. A minor contribution to fire occurrence and area burned are crop fires (Fig. 3g, h), which happen not just in the standing crop but also in harvested cropland. The Netherlands is not alone in this, France and Germany also saw a major spate of crop fires in the extreme drought Summer of 2018 (San-Miguel-Ayanz et al. 2019). Fire can spread rapidly and with high intensity in the dense uniform stands of wheat or corn. Awareness and preparedness for the risk of crop fires in The Netherlands is low, and simple prevention requirements used internationally (e.g. NDSU EXTENSION 2020) such as fire-safe scheduling of harvest timing (e.g. in relation to relative humidity) and fire extinguishers are not in place. With the expected increase in summer drought with climate change, developing policy and educating farmers about this risk is low-hanging fruit.

Finally, the large numbers of fire engines (1492 per year) and water tenders (587 per year) involved in fire suppression illustrate the urban nature of the Dutch wildfire suppression, that relies on the use of heavy vehicles to drive into the field and across the fire site, dousing the flames and the vegetation around it with high volumes of water. The small number of large fires (Fig. 3i) shows that this approach is fairly effective in the Dutch context, and comes at a conservatively estimated cost of 2.4 million € year^−1^ or 5.8 thousand € ha^−1^ (not including all aerial suppression costs). Moreover, significant volumes of water are used in fire suppression. While water use for firefighting is currently not restricted in The Netherlands (in contrast to water use by for e.g. irrigation), the unlimited availability of water for this purpose will unlikely persist in a changing climate. A shift is required to a fire management approach that is aimed at managing fire regimes rather than fighting the flames, focused on fuel management and prevention of impacts before the fire, and use of a toolbox of suppression approaches, including tactical fire, during the fire (Stoof et al. 2020). This shift is crucial to manage fires not just when fires occur under mild weather and on sandy soils that can be driven on, but also when they happen under severe fire weather and in inaccessible terrain like peat bogs. Smarter suppression following international standards such as focusing on understanding fire behavior, assessing spatial and temporal windows for safe and effective fire suppression, tactical fire use, and handcrew techniques may additionally help mitigate negative social-economic impacts by ensuring that crucial emergency services will remain available for e.g. traffic accidents, house fires, and personal emergencies.

### Impacts

The “Costs and resources requested” through to “Fatalities” sections summarized the considerable societal impacts that The Netherlands has incurred as a result of landscape fires, ranging from evacuations of tourists and vulnerable communities to 31 fatalities. Because it is reasonable to assume that we have missed significant fires and fatalities in our record (particularly those in the more distant past), Tables 1 and 2 are not intended as an exhaustive overview but rather to sketch the range of societal impacts incurred. Nevertheless, Table 1 and Fig. 5 do allow verification of news reports that claimed the fire in the Deurnese Peel to be the largest fire in Dutch history, which is likely correct for fires that occurred after 1970 since such large fires would not have gone unnoticed.

The Netherlands is just one example of a range of countries outside Mediterranean Europe where landscape fires pose significant challenges. The most notable recent example were the dozens of fires that occurred simultaneously in the UK in 2022 during the hottest day ever recorded, burning London homes and overwhelming fire services (Walton 2022). Previous examples included the Saddleworth Moor fire (2018) that caused major health concerns because of redistribution of contaminants via air (Graham et al. 2020) and drinking water, and numerous other fires that affected major infrastructure, communities, homes, industry and agriculture, costing several tens of millions of pounds in suppression costs alone (Belcher et al. 2021). In Norway in subzero temperatures, two heathland fires burned 25 km^2^ in 2014, destroying 64 structures (Log et al. 2017). In 2018 alone, wildfires caused 2 deaths and 22 injuries across Austria, Norway, Finland and Sweden, including firefighters (San-Miguel-Ayanz et al. 2019). Table 2 suggests that The Netherlands sees on average two wildfire fatalities every 10 years, one to two orders of magnitude lower than countries like Greece, Portugal and Spain (3–5 per year; Molina-Terrén et al. 2019), Australia (8 per year; Blanchi et al. 2012) and the USA (17 per year; Haynes et al. 2019). Without systematic national and international reporting however, it is likely that these numbers are underestimates (Haynes et al. 2019).

**Fig. 6:**
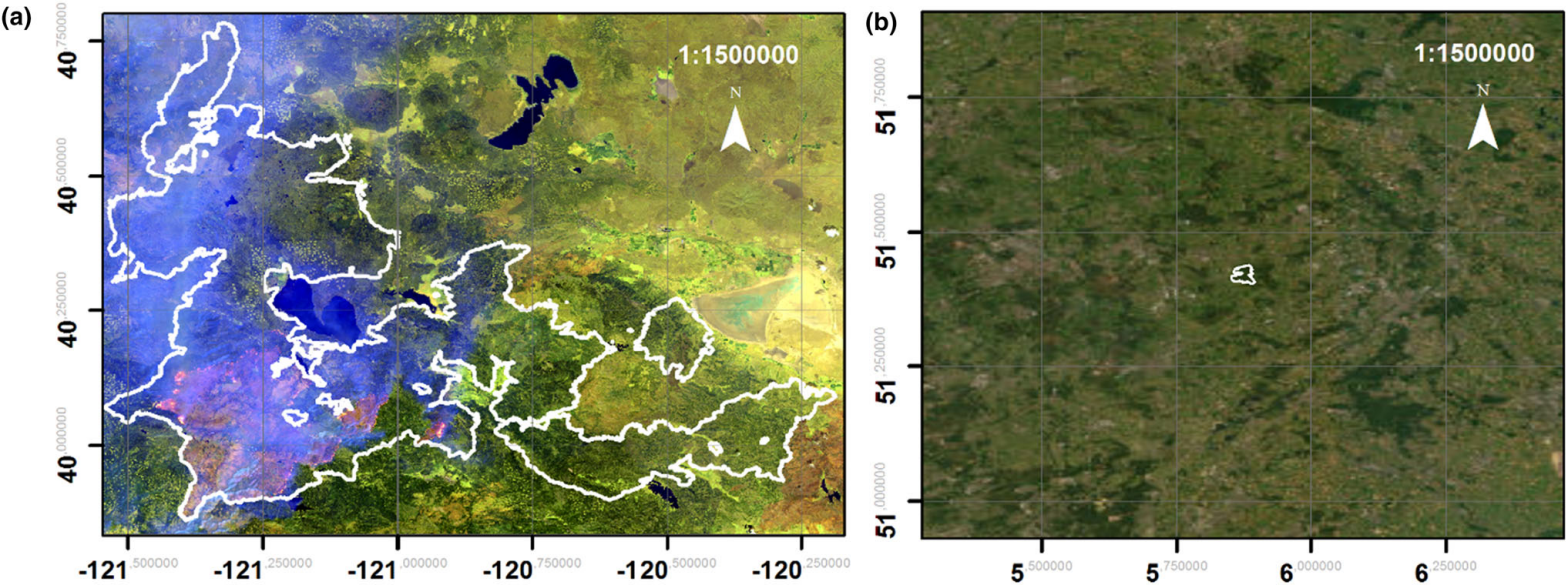
The spatial dimension of landscape fire in The Netherlands compared to that of California, USA, on the same map scale. **a** Dixie fire: (13 July 2021; latitude 39.871306, longitude − 121.389439), burning 389,837 ha before being 100% contained on October 25, 2021. **b** Deurnese Peel fire (20 April 2020, 710 ha). See Appendix S1 for source information for background images

Large fires of up to 710 ha are only a fraction of fires around the world where thousands to millions of hectares burn (e.g. Figure 6 and Andela et al. 2019). Yet as other countries discuss fires in the wildland urban interface or rural urban interface, the densely populated The Netherlands has no wildland; the country is practically only ‘interface’. Like the UK (Belcher et al. 2021), the landscape is highly fragmented with small patch sizes. Even the more rural areas in The Netherlands have a significant population presence as well as vital infrastructure, and on a beautiful sunny day people tend to go outside to much-frequented parks and nature areas for recreation and leisure. While this fragmentation may help slow down fire spread, it does not stop it, as illustrated by the high number of reports of fires jumping roads, trainlines and waterways (Table 1). These landscape features may be important to limiting fire spread, they also play a role in their origin, and in societal vulnerability. Highway delays were calculated at 297 k€ year^−1^, an underestimation given that comparison with Rijkswaterstaat data shows that our database currently fails to capture the majority of small next-to-road fires, traffic data is lacking for roads that are not marked as highway, and smoke disruption from fires not adjacent to highways was not considered. Ultimately, the economic impacts of disruptions depend on factors including the exact timing, duration, location, and extent of the fire, number of people affected, traffic intensity and detouring options. More accurate estimates of wildfire-caused traffic disruptions therefore require inclusion of these data in the database, not only for fires along highways (managed by Rijkswaterstaat) but also along trainlines (ProRail), and ship traffic. A more complete picture of direct and indirect damages can be obtained by also considering costs of any infrastructure failure, business interruption, stress on animals, civilians and aid providers, and damages to buildings and other objects. Discussion within our international network of fire practitioners shows that such comprehensive cost, damages and loss assessments are not carried out, even internationally, which presents a major challenge to proactively develop policy and practice to promote wildfire adaptation and development of fire resilient landscapes and communities.

The general lack of awareness of past and current impacts of fires in The Netherlands and Northwest Europe suggests that ironically they do not happen often enough or are not severe enough to create lasting memories at national or international level. Systematic collection of data on wildfire impacts is therefore essential not only to comprehend the full scale of the challenge, but also to ensure that memory of these events stays alive in the country’s fire culture.

### Need for a legal framework

The data presented here illustrates the wealth of information that can be obtained from dedicated record keeping of landscape fire statistics. This information is crucial for accurate prevention of fire impacts, reduction of undesired ignitions, development of prevention and awareness campaigns, and to design better and fact-based preparedness of civilians, policy makers and emergency services. As such, systematic and long-term time series of fire occurrence is a crucial basis for three pillars of Dutch wildfire management: prevention, research and smart suppression. Furthermore, data on fire regimes are essential as a baseline to assess impacts of climate change, and to fulfill requirements set out under the new EU Forest Strategy (European Commission 2021). Burned area data is moreover required for accurate reporting of land use and forestry greenhouse gas emissions to the UN Climate Convention (UNFCCC) and the Kyoto protocol (Arets et al. 2021). This long list of purposes and legal needs makes it all the more surprising that The Netherlands currently relies on an informal collaboration to record landscape fire statistics. In this light, the decision to halt the 72-year long data series of dedicated ‘forest and nature statistics’, made after a period of mild fire years, is highly unfortunate. A legal framework is needed that prescribes the collection of landscape fire statistics as part of a set of governmental responsibilities to address integrated fire management at national level, coordinated by a single ministry. Much progress has been made in The Netherlands and Northwest Europe regarding wildfire knowledge and preparedness, and that urgency is now felt by many of the actors involved. Yet we observe that because fire is so inter- and transdisciplinary (Stoof and Kettridge 2022) and touches so many different domains and actors, a lack of ownership remains, leaving the topic hanging in many countries. Fire services cannot stop all fires, which the Dutch fire service now publicly acknowledges. Even so there is no legal framework or policy, and initiatives for fuel management, defensible space, resident awareness, and education are local, if at all, with the fire service remaining a largely urban focus in major need of innovation towards smarter suppression (Stoof et al. 2020). Is a disaster needed for true change to be made and ownership taken, like after the deadly 1953 *Watersnoodramp* floods? The Netherlands is world famous for its integrated water management, it is time to learn from its water history (Lambrechts et al. 2023) to develop an integrated fire management strategy using a risk-based approach including assessment of the consequences of failure. This requires systematic data collection and analysis, including a clear institutional framework with roles and responsibilities during all phases of the disaster management cycle: preparedness, response, recovery and mitigation.

## Conclusions and recommendations

With 611 fires and 405 ha burned (Fig. 3), a conservative estimate of 2.7 M€ suppression and restoration costs per year (Fig. 4c), major societal impacts with grave concerns for firefighter safety, and 31 fatalities since 1833, it is safe to say that landscape fires in The Netherlands are not just a future thing: fire is already here. The Dutch fire regime is characterized by a high proportion of small fires, only 2% exceed 10 ha. As such, fires are significantly underreported by EFFIS analyses, that captures only 0.2% of fires and 45% of area burned. This underlines the importance of collecting ground-based data to adequately capture the fire regime and thus its risk to public safety, as every small fire can grow into a big one that even in a fragmented landscape can jump apparent borders and breaks. The high density of the Dutch fire service with rapid response relying on large volumes of water has until now been mostly sufficient to prevent damages, but this will likely change in the future with developments towards more extreme fire behavior that already occur internationally (Castellnou et al. 2022). More complete collection of all fire characteristics in the database will increase the quality of the dataset, with the biggest room for development being the official investigations of fire cause, and consistent and accurate collection of fire locations, size, and fire behavior. Despite a dedicated team being in place, official investigations are scarce. Providing mayors and land owners with information on how they can request an official investigation may help increase their abundance, at the very least for larger, impactful, or recurring fires. Completeness of fire size and vegetation type burned (missing for 62% of fires) may be enhanced by simplifying data entry for local fire departments, but will gain most from field-based mapping of fire perimeters, and fire coordinates. Finally, small fires along highways and railways not needing major fire service assistance are ubiquitous, and despite their small size cause considerable economic damage and social vulnerability. We recommend to systematically collect these data for inclusion in the database like we currently do for the military fires.

Our results highlight the value of ground-based fire counts to accurately capture the existence of fire in the landscape, particularly when fires are small. Given the sizeable underreporting of the EFFIS system (Table 3) we recommend investment in satellite products with closer fly-over times to better capture the scale of the fire challenge in regions with small fires and frequent cloud cover, such as temperate Europe. Given the prevalence of fires in open habitat and the emerging risk of crop fires, we additionally recommend the European Commission to request Member States to report all vegetation fires regardless of the fuel type they burn in, and thus moving away from a sole focus on fire in forests. Finally, systematic national and global recording of wildfire injuries and fatalities, as previously suggested by Haynes et al. (2019) is essential to accurately capture fire impacts on human lives.

To conclude, accurate tracking of the impacts of climate change on fire regimes requires systematic and standardized data collection of fire occurrence, location and cause. We recommend this to be arranged through a legal framework, to ensure fulfillment of international reporting requirements and obtain insights on a topic essential to national public safety. Systematic analysis of societal and economic impacts of fires relies on systematic collection of data on accidents, personal injuries, fatalities, damage to structures and infrastructure, evacuations, physical and mental health, animals, traffic delays, and the number and duration of resources deployed. A solid picture of the fire regime and its impacts is essential to show the urgency of this challenge, and allow development of a fact-based integrated fire management strategy.

### Supplementary Information

Below is the link to the electronic supplementary material.

Supplementary file1 (PDF 683 KB)

## Data Availability

Data and metadata underlying the analyses in this paper are available through Zenodo (10.5281/zenodo.10041816).

## Footnotes

1 www.digibron.nl.

2 www.nos.nl.

3 https://www.brandweernederland.nl/onderwerpen/brandweermonument/.
