# Publisher Correction to: In temperate Europe, fire is already here: The case of The Netherlands

**DOI:** 10.1007/s13280-024-02025-4

**Published:** 2024-05-11

**Authors:** Cathelijne R. Stoof, Edwin Kok, Adrián Cardil Forradellas, Margreet J. E. van Marle

**Affiliations:** 1 Department of Environmental Sciences, Wageningen University, PO Box 47, 6700 AA Wageningen, The Netherlands; 2 Nederlands Instituut Publieke Veiligheid, PO Box 7010, 6801 HA Arnhem, The Netherlands; 3 Technosylva Inc, La Jolla, CA USA; 4 Joint Research Unit CTFC - AGROTECNIO - CERCA, Solsona, Spain; 5 Department of Crop and Forest Sciences, University of Lleida, Lleida, Spain; 6 Deltares, PO Box 177, 2600 MH Delft, The Netherlands

Publisher Correction to: Ambio (2024) 53:604–623 10.1007/s13280-023-01960-y

In the original published article, the data availability statement was incorrectly processed as "Data is obtained from the Dutch national emergency dispatch system GMS, and the Department of Defense" but should have been "Data and metadata underlying the analyses in this paper are available through Zenodo (10.5281/zenodo.10041816)."

The original article has been corrected.

