# Supplementary material for: In temperate Europe, fire is already here: The case of The Netherlands
Source: Ambio. 2024 Feb 5;53(4):604–23. doi: 10.1007/s13280-023-01960-y (PMC10920504; doi:10.1007/s13280-023-01960-y)
Supplement: Supplementary file 1 — Supplementary file1 (PDF 683 KB) [file 13280_2023_1960_MOESM1_ESM.pdf]

Ambio

Supplementary Information

This supplementary information has not been peer reviewed.

Title: **In temperate Europe, fire is already here: the case of The Netherlands**

Appendix S1

Next-to-roadside fires

Fires classified as verge fires or next-to-road fires (*bermbrand*) are not automatically included in the list of wildfires sourced from the Dutch national dispatch system, given that an internal analysis showed that the very large number of next-to-road fires in the dispatch system (>3000 in 2018 alone) often do not concern wildfires but other fires like a burning shrub as a result of a person burning weeds growing between their driveway pavement. Because some of these roadside fires are actual wildfires, we count next-to-road fires in the dispatch system only if the data show that the fire service scaled up the fire and requested more resources upon arrival.

Detailed methodological information regarding fire locations, and classification of fire size, vegetation type and fire cause

- **Location**: location information in the Dutch national emergency dispatch system concerns the name of the village, town or city, as well as the nearest street. For fires that occur away from built up areas the actual location of the burned site may therefore be hundreds of meters or even a few kilometers away from the location registered in the database.
- **Fire size**: because most fires are very small, fire size is not determined through satellite analysis but estimated from photo and video material, or in the field, although this is not systematically done for all fires. For ~60% of fires, size information is not available.
- **Vegetation type**: in line with the estimate of fire size, the vegetation type affected is based on field observation by local fire service personnel, ground-based imagery and news reports, mostly for the larger fires (fires that exceed ~100 m^2). This vegetation type information is listed in two ways: in twelve vegetation classes tailored to the Dutch conditions, as well as using the standard classes in the EU forest fire statistics (forest, other non-wooded, and agriculture; Camia et al., 2014). Note that we do not distinguish between the EU classes 'forest' ('BAFOR'; Camia et al., 2014) and 'other wooded' ('BAOW'; Camia et al., 2014) which requires the fire perimeter with CORINE land cover maps, for which knowledge of the exact location of the burned area is required (personal communication Jesús San Miguel Ayanz). Because satellite-based footprints of the fires are not available, we pragmatically classified all fires in sites with continuous tree cover as forest, thereby combining the EU classes BAFOR and BAOW.
- **Fire cause**: official investigations of fire cause are done by the Dutch Wildfire Cause and Origin Team, police and fire service investigators that completed FI-210 wildfire investigation training,

with results registered following Camia et al. (2013). As most wildfires are not officially investigated, informal assessments of fire cause done by local police or fire departments are separately included in the database.

Quality checks to ensure completeness of data collection

Regarding the data obtained from the Dutch national dispatch system, three main procedures are used to ensure all relevant fires are included in the database. In the Netherlands, fire incidents are reported nationally in the *Gemeenschappelijk Meldkamer Systeem* (GMS), that stores raw data for each incident, and in which the operator assigns labels to fire incidents. GMS is therefore the place where fires are classified as landscape fires or not. Incidents in GSM are publicly communicated in real-time through the so-called P2000 network which is accessible via a range of user interface websites. To populate the database with landscape fires, we manually source relevant incidents from the P2000 network through p2kflex (https://monitor.p2kflex.nl/); usually daily or otherwise weekly. To ensure all relevant fires are captured, an additional social media search is done through OBI4wan, a licensed social media tool. The search term used (in Dutch) is as follows:

> *(natuurbrand OR bosbrand OR "br akker" OR "brand akker" OR akkerbrand OR veenbrand OR duinbrand OR rietbrand OR "brand riet" OR "br riet" OR "br duin" OR "brand veen" OR "brand duin" OR heidebrand OR "brand natuur" OR "br natuur" OR "br heide" OR "br bos" OR "brand bos" OR "brand heide") NOT ("nieuw-zeeland" OR "nieuw zeeland" OR oefening OR ibiza OR frankrijk OR spanje OR italië OR vs OR "verenigde staten" OR usa OR posttype:RETWEET OR australië OR australie OR canada OR madeira OR portugal OR griekenland OR california OR paradise OR californie)*

Since 2020, a second quality check is done through a private Telegram group that automatically screens the P2000 records for relevant fires. Additional information about these fires is subsequently obtained from GMS and from any media coverage summarized by OBI4wan. This may include a possible cause, and information about the possible size (described in the text and/or interpreted from accompanying photo or video material). In the case that public information about a fire is contradicting, an internal fire service quality check is done. Finally, since 2019 a final check of relevant fires is done at the end of each calendar year, when the manually sourced data is cross checked with raw data from GMS. The reason this check is done is to capture fires that were erroneously labeled as a wildfire, as GMS will have the corrected label while errors are not corrected in P2000. On the other hand, GMS may inadvertently contain wildfire trainings, which are removed from our database through this final cross check. Since we did not have

access to GMS in 2017 and 2018 the database may slightly underestimate the total number of fires in those years. Based on our cross checks since 2019 we expect this likely concerns < 25 fires.

In addition to the abovementioned internal steps, since 2021 we also ask the local fire services to report information, as a quality check and also to understand more about the fire itself. This includes information on the vegetation type it burned in, the coordinates of the site and the possible origin, fire size, fire behavior including spot fires, whether the fire jumped any roads, waterways or other fire breaks, and any fire cause and origin investigation that was carried out. This questionnaire is currently sent out via email or embedded in the fire investigation documentation; response rate is moderate at 30% and plans are made to make the questionnaire more accessible, for instance by making it available on the tablets inside each fire engine.

Appendix S2. Results

Comparison of current fire statistics with pre-1994 data

The annual number of fires between 2017 and 2022 was 611 ± 306 (from 212 to 949 fires each year), approximately double the number of wildfires counted between 1945 and 1994 (248 ± 181 fires, ranging from 51 to 904 per year). Because of a higher proportion of fires < 1 ha in the current dataset (89% now, vs. 77% pre-1994), this higher number of fires in the most recent statistics may be explained by the fact that the pre-1994 reporting excluded fires that were stopped when still very small whereas current reporting considers all wildfires regardless of size. Another reason why the current data collection shows larger fire numbers may be that the pre-1994 statistics reportedly underestimated fires outside of forests (IKC, 1995). Regarding burned area, which is often dominated by a small number of very large fires, differences between current data (405 ± 385 ha, from 18 to 1073 ha) and pre-1994 (699 ± 810 ha, from 34 to 3653 ha) were small. Individual fire size of current and past statistics was also similar, with 2% and 2.6% of fires being larger than 10 hectare, respectively (Fig. 2c, 5i).

Background information for Figure 5 and 6 captions

Figure 5. In box 1-5, fire perimeters are outlined on satellite imagery as follows.

1) Deurnese Peel (20 April 2020): fire perimeter on RGB post-fire imagery (Sentinel 2). We also superimposed the fire scar at 10:50:31 UTC time based on Sentinel 2 (bands 12, 11 and 2)
2) ASK 't Harde (18 June 1970), fire perimeter on RGB 2021 basemap (no satellite imagery is available for this date and location. The fire perimeter was delineated based on historical maps.
3) Hoge Veluwe (7 July 1976): fire perimeter on RGB 2021 basemap (no satellite imagery is available for this date and location. The fire perimeter was delineated based on historical maps.
4) Hoge Veluwe (20 April 2014): fire perimeter on RGB post-fire imagery (Landsat 8).
5) Kalmthoutse Heide (21 April 1996): fire perimeter on RGB post-fire imagery (Landsat 5). To determine fire size, we analyzed pre- and post-fire satellite imagery from Landsat to create burn severity maps, from which the burn scar was delineated based on the spectral response of vegetation between both images and fire size was subsequently calculated.

Figure 6. Map A represents the fire scar 2021/07/23 at 18:50 UTC time based on Sentinel 2 (bands 12, 11 and 2).

Table S1 For 40 days between 2017-2022, ≥15 fires occurred; data sorted by date. Three series of (nearly) consecutive days in Aug 2018, Apr 2020 and Sept 2022 are highlighted in bold.

**Date**	**Nr of fires**
30-Jun-18	16
1-Jul-18	31
2-Jul-18	16
4-Jul-18	16
5-Jul-18	16
**15-Jul-18**	**18**
**16-Jul-18**	**21**
**17-Jul-18**	**21**
**18-Jul-18**	**17**
**20-Jul-18**	**16**
**24-Jul-18**	**15**
**25-Jul-18**	**20**
**26-Jul-18**	**27**
**27-Jul-18**	**19**
31-Jul-18	18
5-Aug-18	15
6-Aug-18	21
7-Aug-18	22
20-Apr-19	19
21-Apr-19	15
23-Apr-19	23
5-Apr-20	16
16-Apr-20	15
**20-Apr-20**	**19**
**21-Apr-20**	**17**
**22-Apr-20**	**23**
**23-Apr-20**	**15**
**24-Apr-20**	**20**
31-May-20	19

1-Jun-20	19
18-Jul-22	23
19-Jul-22	21
20-Jul-22	17
9-Aug-22	15
12-Aug-22	18
13-Aug-22	17
25-Aug-22	16
**1-Sep-22**	**23**
**2-Sep-22**	**21**
**3-Sep-22**	**21**

Table S2 Coordinates and source information for the historical record of significant fires in the Netherlands since 1970

When	Where	Latitude	Longitude	Source
18 Jun 1970	ASK	52.4063	5.9348	(Broekman, 1985; IKC, 1970)
7 Jul 1976	Hoge Veluwe	52.0439	5.9802	(Lavèn, 1976)<br>https://nos.nl/artikel/638192-grootste-brand-op-veluwe-ooit<br>https://www.rizoomes.nl/brandweer/bosbrand-roosendaalse-veld/<br>https://www.gld.nl/nieuws/2405933/dit-doet-burgers-zoo-als-de-dierentuin-wordt-bedreigd-door-een-natuurbrand

8 Jul 1976	Leenderheide	51.3522	5.5227	https://www.heiheghoogeind.nl/bosbrand-leenderbos1976/
31 May 1977	Best	51.5078	5.4005	brandweer-monument
12 May 1980	Doldersum	52.9002	6.2569	https://www.digibron.nl/viewer/collectie/Digibron/offset/0/zoekwoord/camping+Sonnekamp+in+Vledder+brand/id/tag:RD.nl,19800513:newsml_42b284de39670803541c0394365d3481<br>Schade heidebrand 1980 hersteld Doldersumseveld is weer als vanouds. "Nieuwsblad van het Noorden". Groningen, 08-12-1982, p. 3. Geraadpleegd op Delpher op 03-08-2022, https://resolver.kb.nl/resolve?urn=ddd:011010557:mpeg21:p003
23 May 1980	Herkenbosch	51.1783	6.0572	https://www.digibron.nl/viewer/collectie/Digibron/offset/0/zoekwoord/brand+naaldbos/id/tag:RD.nl,19800527:newsml_08ef8e003d6ac11b094e3245f6570d3f

15 May 1980	Deurnesche Peel	51.4244	5.8843	https://www.digibron.nl/viewer/collectie/Digibron/offset/5/zoekwoord/%28brand+peel%29/id/tag:RD.nl,19800516:newsml_491706bb36dd71053d97c9fac7295187
15 May 1980	Hoog Soeren	52.1929	5.8508	https://www.digibron.nl/viewer/collectie/Digibron/offset/5/zoekwoord/%28brand+peel%29/id/tag:RD.nl,19800516:newsml_491706bb36dd71053d97c9fac7295187
24 Apr 1982	Arnhemse Heide	52.0499	5.9205	https://krantenbankzeeland.nl/issue/pzc/1982-04-26/edition/0/page/18
28 Apr 1984	Sprengenberg (Hellendoorn)	52.3474	6.4227	https://krantenbankzeeland.nl/issue/pzc/1984-04-30/edition/0/page/3
18 Jun 1986	Someren	51.3631	5.6616	https://www.digibron.nl/viewer/collectie/Digibron/offset/7/zoekwoord/%28heidebrand%29/id/tag:RD.nl,19860619:newsml_a727e584c160b5844bb725969c1fc1a3
10 May 1987	Witterveld (Havelte)	52.9622	6.5091	https://www.digibron.nl/viewer/collectie/Digibron/offset/8/zoekwoord/%28heidebrand%29/id/tag:RD.nl,19870511:newsml_bfe7e6c5223cc6917c80bc6304def345

27 Jul 1989	Beekhuizerzand	52.3336	5.6772	https://www.brandweernederland.nl/onderwerpen/brandweermonument/
23 Apr 1994	Sallandse Heuvelrug	52.3387	6.4241	https://www.digibron.nl/viewer/collectie/Digibron/id/tag:RD.nl,19940425:newsml_29a3ef7c9eb0b6aa579ad6cbfbecac0c<br>Vuurzee verwoest Sallandse heuvelrug. "Nieuwsblad van het Noorden". Groningen, 25-04-1994, p. 5. Geraadpleegd op Delpher op 05-08-2022, https://resolver.kb.nl/resolve?urn=ddd:011005069:mpeg21:p005

11 Aug 1995	Kootwijk, A1	52.1957	5.7441	Ruim honderd hectare natuur verwoest door bermbrand langs A1 Van onze verslaggever AMSTERDAM. "De Volkskrant". 's-Hertogenbosch, 12-08-1995. Geraadpleegd op Delpher op 05-08-2022, https://resolver.kb.nl/resolve?urn=ABCDDD:010870939:mpeg21:p001<br>Brand legt honderden hectaren natuurgebied in as. "Trouw". Meppel, 12-08-1995. Geraadpleegd op Delpher op 05-08-2022, https://resolver.kb.nl/resolve?urn=ABCDDD:010822547:mpeg21:p003<br>A1 dicht. "Algemeen Dagblad". Rotterdam, 12-08-1995. Geraadpleegd op Delpher op 05-08-2022, https://resolver.kb.nl/resolve?urn=KBPERS01:003130010:mpeg21:p00001<br>https://www.digibron.nl/viewer/collectie/Digibron/offset/1/zoekwoord/%28kootwijk+brand%29/id/tag:RD.nl,19950812:newsml_19ff52b41431781e5797496c796b185d376c7ec7c72de61617537549

20 Apr 1996	Hooge en Lage Mierde	51.4350	5.1318	https://www.digibron.nl/viewer/collectie/Digibron/offset/0/zoekwoord/landgoed+de+utrecht+brand/id/tag:RD.nl,19960422:newsml_3ef169da9e21f291748d4132d19d329dffa55ff77479b640ce8d1c28
20 Apr 1996	Loonse en Drunense Duinen	51.6535	5.1289	https://www.digibron.nl/viewer/collectie/Digibron/offset/0/zoekwoord/landgoed+de+utrecht+brand/id/tag:RD.nl,19960422:newsml_3ef169da9e21f291748d4132d19d329dffa55ff77479b640ce8d1c28
21 Apr 1996	Kalmthoutse Heide	51.4018	4.4151	https://pureportal.inbo.be/portal/files/821446/Sioen_1997_OverzichtBos_HeidebrandenVlaamseGewest.pdf https://www.omroepbrabant.nl/nieuws/831560/brand-kalmthoutse-heide-fotos-en-video https://www.digibron.nl/viewer/collectie/Digibron/offset/8/zoekwoord/kalmthoutse+heide+/id/tag:RD.nl,19960422:newsml_70d00db8a2eeb55fac0e7aa596f3691169f3915888354755e485978b
20 Apr 1997	Kalmthoutse Heide	51.4018	4.4151	https://www.digibron.nl/viewer/collectie/Digibron/offset/2/zoekwoord/kalmthoutse+heide+/id/tag:RD.nl,19970422:newsml_4eb772d140a17e78a48df7f77b44aa3f782af0c6d4e457e7c7562231

28 Apr 1999	Rozendaal	52.0566	5.9415	https://www.digibron.nl/viewer/collectie/Digibron/offset/0/zoekwoord/rozendaal+brand/id/tag:RD.nl,19990429:newsml_f5c18c46a047e8b03f57ccc0193b918f
13 May 2001	Loonse en Drunense Duinen	51.6535	5.1289	https://www.digibron.nl/viewer/collectie/Digibron/offset/0/zoekwoord/loonse+drunense+duinen+brand/id/tag:RD.nl,20010514:newsml_57654b55bdbba1eee54af087ded324ae
17 Apr 2003	ASK	52.4014	5.9065	https://www.digibron.nl/viewer/collectie/Digibron/offset/5/zoekwoord/%28heidebrand%29/id/tag:RD.nl,20030418:newsml_3fc6a5f2d0924185ebb8065daa0267f9
25 Aug 2003	Utrecht	52.0983	5.0874	https://www.digibron.nl/viewer/collectie/Digibron/offset/2/zoekwoord/hoge+veluwe+brand/id/tag:RD.nl,20030826:newsml_678c8cf52d63b39e1ec7354997e5305c
15 May 2004	Terschelling	53.4305	5.4671	https://www.digibron.nl/viewer/collectie/Digibron/offset/0/zoekwoord/terschelling+brand/id/tag:RD.nl,20040517:newsml_5bade9f03483632b9213d344a2089461
6 May 2006	Hoog Soeren	52.2386	5.7941	https://www.digibron.nl/viewer/collectie/Digibron/offset/3/zoekwoord/bosbrand/id/tag:RD.nl,20060508:newsml_98ac1643783ff2dae82ccff4db28a7ce

9 Jul 2006	Hoge Veluwe	52.0789	5.8325	https://www.digibron.nl/viewer/collectie/Digibron/offset/0/zoekwoord/hoge+veluwe+brand/id/tag:RD.nl,20060710:newsml_b87f3d166c8edc7a1b38680a22d105b4 https://www.digibron.nl/viewer/collectie/Digibron/offset/1/zoekwoord/hoge+veluwe+brand/id/tag:RD.nl,20060711:newsml_93135f79a68d247be1a0961085c0a7c0
29 Apr 2007	Ermelo	52.2614	5.7365	https://www.digibron.nl/viewer/collectie/Digibron/offset/1/zoekwoord/ermelo+brand/id/tag:RD.nl,20070501:newsml_56f8d0acebd00b09e8ef2e8e2b1909e3
28 Aug 2009	Schoorl	52.7068	4.6799	https://www.nhnieuws.nl/nieuws/251859/vandaag-tien-jaar-geleden-hoe-het-vuur-schoorl-op-een-paar-meter-naderde

16 Sep 2009	Bergen	52.6697	4.6814	https://www.nhnieuws.nl/nieuws/251859/vandaag-tien-jaar-geleden-hoe-het-vuur-schoorl-op-een-paar-meter-naderde
24 Apr 2009	Hoog Soeren	52.2004	5.8366	https://www.digibron.nl/viewer/collectie/Digibron/offset/0/zoekwoord/brand+assel/id/tag:RD.nl,20090425:newsml_373623dd2cc4bb0602b1a4e5bf4c2a1e
27 Apr 2009	Wierdense Veld	52.3799	6.5287	https://www.digibron.nl/viewer/collectie/Digibron/offset/1/zoekwoord/wierdense+veld+brand/id/tag:RD.nl,20090427:newsml_e352c07e268415875fbf7a1b519b72a0
14 Apr 2010	Bergen	52.6650	4.6383	https://www.nhnieuws.nl/nieuws/251859/vandaag-tien-jaar-geleden-hoe-het-vuur-schoorl-op-een-paar-meter-naderde<br>https://www.parool.nl/nieuws/bergen-aan-zee-ontruimd-wegens-duinbrand~b40406df/?referrer=https%3A%2F%2Fwww.google.com%2F<br>https://www.parool.nl/nieuws/bergen-aan-zee-verwacht-windkracht-7~bded5426<br>https://www.digibron.nl/viewer/collectie/Digibron/offset/0/zoekwoord/brand+bergen+aan+zee/id/tag:RD.nl,20100415:newsml_ac049b11ca8b8592d28397b18ded7a94

2 Jul 2010	Strabrechtse Heide	51.4038	5.6122	https://archief.nipv.nl/wp-content/uploads/sites/2/2022/03/201102-IOOV-Brand-Strabrechtse-Heide-Deel-1-hoofdstructuur.pdf https://archief.nipv.nl/wp-content/uploads/sites/2/2022/03/201102-IOOV-Brand-Strabrechtse-Heide-Deel-2-bestrijding-natuurbrand.pdf
20 Apr 2010	Hoog Soeren	52.1925	5.8522	https://www.digibron.nl/viewer/collectie/Digibron/offset/1/zoekwoord/hoog+soeren+brand/id/tag:RD.nl,20100421:newsml_04db7a9d41e1f93c578dc2ff9f85128f
1 May 2011	Schoorl	52.7068	4.6799	https://www.digibron.nl/viewer/collectie/Digibron/offset/0/zoekwoord/brand+schoorl/id/tag:RD.nl,20110502:newsml_fff9d76c831dee91158337f7bcbb8b5a https://www.digibron.nl/viewer/collectie/Digibron/offset/1/zoekwoord/brand+schoorl/id/tag:RD.nl,20110505:newsml_2f30c6ca7bf590525446e59e6db28a43

24 Apr 2011	Fochteloërveen	53.0161	6.4099	https://www.digibron.nl/viewer/collectie/Digibron/offset/3/zoekwoord/brand+fochteloerveen/id/tag:RD.nl,20110426:newsml_0119a6c3ae60d0cbbeaaf0d232caf0f5
3 Jun 2011	Aamsveen	52.1833	6.9519	https://www.digibron.nl/viewer/collectie/Digibron/offset/1/zoekwoord/aamsveen+brand/id/tag:RD.nl,20110606:newsml_9b82c628bb798ea8b028434ef898e6e1 https://www.digibron.nl/viewer/collectie/Digibron/offset/2/zoekwoord/aamsveen+brand/id/tag:RD.nl,20110604:newsml_b388d542555885c964c10debe0e558a1
25 May 2011	Kalmthoutse Heide	51.4018	4.4151	https://www.omroepbrabant.nl/nieuws/847563/brand-kalmthoutse-heide-onbeheersbaar-door-problemen-brandweer https://www.standaard.be/cnt/dmf20110527_004
1 Apr 2012	Radio Kootwijk	52.1689	5.8252	https://www.digibron.nl/viewer/collectie/Digibron/offset/0/zoekwoord/kootwijk+brand/id/tag:RD.nl,20120402:newsml_e0fe0400672104c881b93aa4665d4093
20 Apr 2014	Hoge Veluwe	52.0874	5.8471	https://www.hogeveluwe.nl/nl/nieuws/2014-04-24-brand-treft-minder-dan-10-van-het-park

17 Apr 2014	Mastbos	51.5453	4.7610	https://www.omroepbrabant.nl/nieuws/1829075/brand-verwoest-deel-mastbos-breda-jeugdgevangenen-den-hey-acker-overgebracht-naar-de-koepel
13 Mar 2015	Veenhuizen	53.0161	6.4099	https://nos.nl/artikel/2024542-forse-natuurbrand-in-drenthe-ruim-100-hectare-afgebrand
17 May 2015	Chaam	51.5168	4.8910	https://www.omroepbrabant.nl/nieuws/2095093/zeer-grote-brand-in-bossen-chaam-sein-brand-meester-60-a-70-hectare-afgebrand
18 Jul 2015	Leenderheide	51.3695	5.5062	https://www.omroepbrabant.nl/nieuws/2128314/zes-hectare-leenderheide-verwoest-door-brand-brabants-landschap-houdt-gebied-in-de-gaten
22 Jun 2017	Maria Peel	51.4277	5.8931	https://www.nu.nl/binnenland/4788288/natuurgebied-in-brabant-beschadigd-flinke-natuurbrand.html
7 Jul 2017	Malpie	51.3183	5.4406	https://www.ed.nl/valkenswaard/grote-brand-in-natuurgebied-de-malpie-in-valkenswaard-redelijk-onder-controle~a70fb659/

30 Jun 2018	Wedde	53.0779	7.0611	https://www.oldambtnu.nl/zeer-grote-brand-bedreigt-camping-weddebergen<br>https://www.hartvannederland.nl/nieuws/brandend-graanveld-bedreigt-naastgelegen-camping-wedde
7 Aug 2018	Wateren	52.9146	6.2929	https://www.rtvdrenthe.nl/nieuws/150547/75-hectare-natuur-veranderde-in-een-maanlandschap-zo-verliep-de-brand-in-wateren<br>https://www.rtvdrenthe.nl/nieuws/137597/zeer-grote-heidebrand-in-wateren-campings-ontruimd
16 Jul 2018	Heemskerk	52.5165	4.5958	https://www.vrk.nl/Nieuws/2018/07/16/Duinbrand-bij-Heemskerk<br>internal fire service records
18 Jul 2018	Drunen	51.6535	5.1289	https://www.omroepbrabant.nl/nieuws/2813368/grote-brand-in-loonse-en-drunense-duinen-luchtmacht-zet-twee-chinooks-in-om-te-blussen<br>internal fire service records

15 Jul 2018	ASK	52.4014	5.9065	https://www.rtlnieuws.nl/nieuws/nederland/artikel/4289366/chinook-ingezet-bij-grote-heidebrand-oldebroek<br>https://www.rtvfocuszwolle.nl/grote-rookwolk-te-zien-in-zwolle-door-brand-op-defensieterrein-t-harde/amp/<br>internal fire service records
1 Jul 2018	Budel	51.2560	5.5885	https://www.omroepbrabant.nl/nieuws/2799042/groot-stuk-grond-in-budel-verwoest-natuurbrand-onder-controle
27 Feb 2018	Deurnese Peel	51.4277	5.8931	https://www.omroepbrabant.nl/nieuws/2654433/gebiedsverbod-in-deurnese-peel-brand-beslaat-oppervlakte-van-40-voetbalvelden<br>https://www.1limburg.nl/nieuws/1368157/groot-gebied-getroffen-door-brand-helenaveen
8 Apr 2019	Hilversum	52.2515	5.1818	internal fire service records
10 Apr 2019	Arnhemse Heide	52.0488	5.9207	https://nos.nl/artikel/2279856-natuurbrand-bij-arnhem-onder-controle-rook-is-weggetrokken

15 May 2019	Leusderheide	52.1047	5.3418	https://nos.nl/artikel/2284761-grote-brand-op-militair-oefenterrein-bij-leusden
20 Apr 2020	Deurnese Peel	51.4277	5.8931	(Stoof et al., 2020)

20 Apr 2020	De Meinweg	51.1696	6.1294	(Instituut Fysieke Veiligheid, 2020)
21 Apr 2020	Moergestel	51.5410	5.1520	https://nos.nl/artikel/2331179-natuurbranden-in-de-meinweg-en-deurnese-peel-nog-niet-onder-controle
31 Mar 2021	Sint Jansklooster	52.6798	6.0224	https://www.destentor.nl/steenwijkerland/slachtoffer-rietbrand-sint-jansklooster-is-80-jarige-vrouw-uit-steenwijkerland~a26236f5/

14 Apr 2022	Putte	51.3592	4.3965	internal fire service records
19 Jul 2022	ASK 't Harde	52.4080	5.9458	internal fire service records
9 Aug 2022	Ouddorp	51.7838	3.8736	https://nos.nl/artikel/2439926-grote-duinbrand-bij-brouwersdam-onder-controle<br>internal fire service records
31 Aug 2022	Mariapeel	51.4172	3.8736	https://www.boswachtersblog.nl/limburg/2022/09/28/na-de-brand-in-de-mariapeel/

Additional references

Broekman, J. (1985). *Grote branden in de lage landen*.
Camia, A., DURRANT, H.T., San-Miguel-Ayanz, J. (2013). *Harmonized classification scheme of fire causes in the EU adopted for the European Fire Database of EFFIS*. European Commission.
Camia, A., Durrant, T., San-Miguel-Ayanz, J. (2014). The European fire database: technical specifications and data submission. *EUR 26546 EN. Luxemburg (Luxemburg), Publications Office of the European Union*.
IKC. (1970). Statistiek van bos- en heidebranden in 1970. In: Staatsbosbeheer, afdeling Statistiek.
IKC. (1995). *Statistiek van branden in bos- en natuurterrein 1993* (Werkdocument IKC Natuurbeheer Nr. 75, Issue.
Instituut Fysieke Veiligheid. (2020). *Natuurbrand in De Meinweg en de evacuatie van Herkenbosch. Een evaluatie in opdracht van Veiligheidsregio Limburg-Noord*. https://nipv.nl/wp-content/uploads/2022/02/20201013-IFV-Natuurbrand-in-De-Meinweg-en-de-evacuatie-van-Herkenbosch.pdf
Lavèn, J. (1976). Grote bosbrand bij Arnhem bewees noodzaak van regionale inzet. In (Vol. 30, pp. 149-152). De Brandweer.
Stoof, C.R., Tapia, V.M., Marcotte, A.L., Stoorvogel, J.J., Ribau, M.C. (2020). *Relatie tussen natuurbeheer en brandveiligheid in de Deurnese Peel: onderzoek naar aanleiding van de brand in de Deurnese Peel van 20 april 2020*.
